# Supplementary material for: Evolving MRSA: High-level β-lactam resistance in Staphylococcus aureus is associated with RNA Polymerase alterations and fine tuning of gene expression
Source: PLoS Pathog. 2020 Jul 24;16(7):e1008672. doi: 10.1371/journal.ppat.1008672 (PMC7380596; doi:10.1371/journal.ppat.1008672)
Supplement: S1 Table — *, denotes trained strains with intermediate oxacillin resistance (TI); †, denotes trained strains with high-level oxacillin resistance (TR); ‡, denotes TI strain trained further for high-level oxacillin resistance (TIR). (PDF) [file ppat.1008672.s001.pdf]

Strain	Methicillin gradient plate	Oxacillin MIC
SH1000		0.12 µg/ml
**Parental strain SH1000**		
lysA::p*mecA* (SJF4996)		2 µg/ml
**Parental strain *lysA*::p*mecA* (SJF4996)**		
lysA::p*mecA*-TI1* (SJF4998)	0-5 µg/ml methicillin	2 µg/ml
lysA::p*mecA*-TI2* (SJF4999)	0-5 µg/ml methicillin	16 µg/ml
lysA::p*mecA*-TI3* (SJF5001)	0-5 µg/ml methicillin	16 µg/ml
lysA::p*mecA*-TI4* (SJF5002)	0-20 µg/ml methicillin	16 µg/ml
**Parental strain *lysA*::p*mecA*-TI1 (SJF4998)**		
lysA::p*mecA*-TIR1‡ (SJF5006)	0-20 µg/ml methicillin	≥256 µg/ml
lysA::p*mecA*-TIR2‡ (SJF5007)	0-20 µg/ml methicillin	≥256 µg/ml
lysA::p*mecA*-TIR3‡ (SJF5008)	0-20 µg/ml methicillin	≥256 µg/ml
**Parental strain *lysA*::p*mecA* (SJF4996)**		
lysA::p*mecA*-TR1† (SJF5000)	0-5 µg/ml methicillin	≥256 µg/ml
lysA::p*mecA*-TR2† (SJF5003)	0-20 µg/ml methicillin	≥256 µg/ml
lysA::p*mecA*-TR3† (SJF5004)	0-20 µg/ml methicillin	≥256 µg/ml
lysA::p*mecA*-TR4† (SJF5005)	0-20 µg/ml methicillin	≥256 µg/ml
lysA::p*mecA*-TR5† (SJF5031)	0-5 µg/ml methicillin	≥256 µg/ml
lysA::p*mecA*-TR6† (SJF5032)	0-5 µg/ml methicillin	≥256 µg/ml
lysA::p*mecA*-TR7† (SJF5033)	0-5 µg/ml methicillin	≥256 µg/ml
lysA::p*mecA*-TR8† (SJF5034)	0-5 µg/ml methicillin	≥256 µg/ml
**Parental strain *lysA*::p*mecA*-TR2† (SJF5003)**		
lysA::kan (p*mecA*-cured) (SJF5010)		0.5 µg/ml
**Parental strain *lysA::kan* (p*mecA*-cured) (SJF5010)**		
lysA::p*mecA*+ (SJF5011)		≥256 µg/ml

S1 Table: List of *S. aureus* strains associated with single copy *mecA* (*lysA*::p*mecA*).
*, denotes trained strains with intermediate oxacillin resistance (TI); †, denotes trained strains with high-level oxacillin resistance (TR); ‡, denotes TI strain trained further for high-level oxacillin resistance (TIR).
