# Supplementary material for: Evolving MRSA: High-level β-lactam resistance in Staphylococcus aureus is associated with RNA Polymerase alterations and fine tuning of gene expression
Source: PLoS Pathog. 2020 Jul 24;16(7):e1008672. doi: 10.1371/journal.ppat.1008672 (PMC7380596; doi:10.1371/journal.ppat.1008672)
Supplement: S2 Table — *, Only DNA sequencing was performed for the rpoB and rpoC genes; †, frameshift mutation; ‡, 11-bp insertion; a, representative strains used in this study; b, data taken from [1]; S, Sensitive. OX, Oxacillin; FOX, Cefoxitin; PG, Penicillin G; RIF, Rifampicin. (PDF) [file ppat.1008672.s002.pdf]

Strain/Genome position		MIC (μg/ml)				Nucleotide change	Amino acid Change	Locus tag (Gene)	Protein
		OX	FOX	PG	RIF				
**Parental strain SH1000**[a]		0.12	2	0.064	S				
***lysA*::p*mecA* (SJF4996)**[a]		2	24	0.75	S				
**Parental strain *lysA*::p*mecA* (SJF4996)**									
**TI1 (SJF4998)**		2	24	0.75	S				
**TI2 (SJF4999)**	525575	16	24	2	S	G-A	G1139D	SAOUHSC_00524 (*rpoB*)	DNA-directed RNA polymerase subunit β
**TI3 (SJF5001)**	529097	16	24	1.5	S	A-T	I1084F	SAOUHSC_00525 (*rpoC*)	DNA-directed RNA polymerase subunit β'
**TI4 (SJF5002)**	290628	16	16	1	S	T-G	S169A	SAOUHSC_00270	Hypothetical protein
	528402					C-A	S852Y	SAOUHSC_00525 (*rpoC*)	DNA-directed RNA polymerase subunit β'
**TR1 (SJF5000)**	528062	≥256	≥256	8	S	C-A	R739S	SAOUHSC_00525 (*rpoC*)	DNA-directed RNA polymerase subunit β'
	590401[†]					CG-C	R189	SAOUHSC_00591	Hypothetical protein
**TR2 (SJF5003)**[a]	524946	≥256	≥256	12	S	T-A	H929Q	SAOUHSC_00524 (*rpoB*)	DNA-directed RNA polymerase subunit β
	590401[†]					CG-C	R189	SAOUHSC_00591	Hypothetical protein
	2134372[‡]					A-AAGCCTTTAACG		SAOUHSC_02301 (*rsbU*)	Sigma B regulatory protein
lysA::kan* (SJF5010)[a]	524946	0.25	4	0.094	S	T-A	H929Q	SAOUHSC_00524 (*rpoB*)	DNA-directed RNA polymerase subunit β
**TR3 (SJF5004)**	524946	≥256	≥256	6	S	T-A	H929Q	SAOUHSC_00524 (*rpoB*)	DNA-directed RNA polymerase subunit β
	590401[†]					CG-C	R189	SAOUHSC_00591	Hypothetical protein
**TR4 (SJF5005)**	528644	≥256	≥256	16	S	G-C	E933Q	SAOUHSC_00525 (*rpoC*)	DNA-directed RNA polymerase subunit β'
**TR5 (SJF5031)***	524087	≥256	≥256	12	S	A-C	Q643P	SAOUHSC_00524 (*rpoB*)	DNA-directed RNA polymerase subunit β
**TR6 (SJF5032)***	524087	≥256	≥256	12	S	A-C	Q643P	SAOUHSC_00524 (*rpoB*)	DNA-directed RNA polymerase subunit β
**TR7 (SJF5033)***	524087	≥256	≥256	8	S	A-C	Q643P	SAOUHSC_00524 (*rpoB*)	DNA-directed RNA polymerase subunit β
**TR8 (SJF5034)***[a]	528065	≥256	≥256	12	S	G-C	G740R	SAOUHSC_00525 (*rpoC*)	DNA-directed RNA polymerase subunit β'
**Parental strain *lysA*::p*mecA*-TI1 (SJF4998)**									
**TIR1 (SJF5006)**	289906	≥256	≥256	8	S	T-A	L161Q	SAOUHSC_00269	Hypothetical protein
	528059					G-A	A738T	SAOUHSC_00525 (*rpoC*)	DNA-directed RNA polymerase subunit β'
	2134372[‡]					A-AAGCCTTTAACG		SAOUHSC_02301 (*rsbU*)	Sigma B regulatory protein
**TIR2 (SJF5007)**	528695	≥256	≥256	12	S	G-C	G950R	SAOUHSC_00525 (*rpoC*)	DNA-directed RNA polymerase subunit β'
**TIR3 (SJF5008)**	524074	≥256	≥256	16	S	G-T	G639C	SAOUHSC_00524 (*rpoB*)	DNA-directed RNA polymerase subunit β
	525004					G-C	D949H	SAOUHSC_00524 (*rpoB*)	DNA-directed RNA polymerase subunit β
**MRSA isolates**									
COL*		≥256	≥256	32	S		A798V<br>S875L	SAOUHSC_00524 (*rpoB*)	DNA-directed RNA polymerase subunit β
MRSA252*		≥256	≥256	≥256	S		Y737F	SAOUHSC_00524 (*rpoB*)	DNA-directed RNA polymerase subunit β
							I864V	SAOUHSC_00525 (*rpoC*)	DNA-directed RNA polymerase subunit β'
Mu50*		≥256	≥256	24	≥256		H481Y	SAOUHSC_00524 (*rpoB*)	DNA-directed RNA polymerase subunit β

Mu3*	≥256	≥256	48	S			
USA300_FPR3757*	1	16	0.25	S	R857H	SAOUHSC_00525 (*rpoC*)	DNA-directed RNA polymerase subunit β’
MW2*	8	32	32	S			
					D471Y	SAOUHSC_00524 (*rpoB*)	DNA-directed RNA polymerase subunit β
					A473S	SAOUHSC_00524 (*rpoB*)	DNA-directed RNA polymerase subunit β
JH9[b]*	0.75			16	A477S	SAOUHSC_00524 (*rpoB*)	DNA-directed RNA polymerase subunit β
					E478D	SAOUHSC_00524 (*rpoB*)	DNA-directed RNA polymerase subunit β
					E854K	SAOUHSC_00525 (*rpoC*)	DNA-directed RNA polymerase subunit β’

References:

1. Mwangi MM, Wu SW, Zhou Y, Sieradzki K, de Lencastre H, Richardson P, et al. Tracking the in vivo evolution of multidrug resistance in *Staphylococcus aureus* by whole-genome sequencing. Proc Natl Acad Sci. 2007;104(22):9451–6.

S2 Table: Identification of mutations and Antibiogram of MRSA and SH1000 derived oxacillin resistant strains.
*, Only DNA sequencing was performed for the *rpoB* and *rpoC* genes; †, frameshift mutation; ‡, 11-bp insertion; [a], representative strains used in this study; [b], data taken from [1]; S, Sensitive. OX, Oxacillin; FOX, Cefoxitin; PG, Penicillin G; RIF, Rifampicin
