# Supplementary material for: Evolving MRSA: High-level β-lactam resistance in Staphylococcus aureus is associated with RNA Polymerase alterations and fine tuning of gene expression
Source: PLoS Pathog. 2020 Jul 24;16(7):e1008672. doi: 10.1371/journal.ppat.1008672 (PMC7380596; doi:10.1371/journal.ppat.1008672)
Supplement: S3 Table — *, denotes trained strains with intermediate oxacillin resistance (TI); †, denotes trained strains with high-level oxacillin resistance (TR); ‡, denotes TI strain trained further for high-level oxacillin resistance (TIR). (PDF) [file ppat.1008672.s003.pdf]

Strain	Methicillin gradient plate	Oxacillin MIC
SH1000		0.12 µg/ml
**Parental strain SH1000**		
pRB474-p*mecA* (SJF4981)		0.25 µg/ml
**Parental strain pRB474-p*mecA* (SJF4981)**		
pRB474-p*mecA*-TI1* (SJF4984)	0-5 µg/ml methicillin	2 µg/ml
pRB474-p*mecA*-TI2* (SJF4989)	0-5 µg/ml methicillin	2 µg/ml
pRB474-p*mecA*-TI3* (SJF4992)	0-5 µg/ml methicillin	4 µg/ml
pRB474-p*mecA*-TI11* (SJF5194)	0-5 µg/ml methicillin	16 µg/ml
**Parental strain pRB474-p*mecA*-TI1* (SJF4984)**		
pRB474-p*mecA*-TIR1‡ (SJF4986)	0-20 µg/ml methicillin	≥256 µg/ml
pRB474-p*mecA*-TIR2‡ (SJF4987)	0-20 µg/ml methicillin	≥256 µg/ml
pRB474-p*mecA*-TIR3‡ (SJF4988)	0-20 µg/ml methicillin	≥256 µg/ml
**Parental strain pRB474-p*mecA* (SJF4981)**		
pRB474-p*mecA*-TR1† (SJF4985)	0-5 µg/ml methicillin	≥256 µg/ml
pRB474-p*mecA*-TR2† (SJF4990)	0-5 µg/ml methicillin	≥256 µg/ml
pRB474-p*mecA*-TR3† (SJF4991)	0-5 µg/ml methicillin	≥256 µg/ml
**Parental strain pRB474-p*mecA*-TR3† (SJF4991)**		
pRB474-p*mecA* cured (SJF4993)		0.5 µg/ml
**Parental strain pRB474-p*mecA* cured (SJF4993)**		
pRB474-p*mecA*$^+$ (SJF4995)		≥256 µg/ml

S3 Table: List of *S. aureus* strains associated with plasmid-borne *mecA* (pRB474 p*mecA*).

*, denotes trained strains with intermediate oxacillin resistance (TI); †, denotes trained strains with high-level oxacillin resistance (TR); ‡, denotes TI strain trained further for high-level oxacillin resistance (TIR).
