# Supplementary material for: Evolving MRSA: High-level β-lactam resistance in Staphylococcus aureus is associated with RNA Polymerase alterations and fine tuning of gene expression
Source: PLoS Pathog. 2020 Jul 24;16(7):e1008672. doi: 10.1371/journal.ppat.1008672 (PMC7380596; doi:10.1371/journal.ppat.1008672)
Supplement: S4 Table — (PDF) [file ppat.1008672.s004.pdf]

Genome position		Oxacillin MIC (μg/ml)	Nucleotide change	Amino acid Change	Locus tag (Gene)	Protein
**Parental strain SH1000 (SJF682)**						
**pRB474-p*mecA* (SJF4981)**		0.25				
**Parental strain pRB474-p*mecA* (SJF4981)**						
**TI1 (SJF4984)**	9045		C-T	R681C	SAOUHSC_00006 (*gyrA*)	DNA gyrase subunit A
	176626	2	G-A	D784N	SAOUHSC_00162 (*hsdR*)	Type I site-specific deoxyribonuclease
	2698103		C-T	S511L	SAOUHSC_02932 (*betA*)	Oxygen-dependent choline dehydrogenase
**TI2 (SJF4989)**	9045	2	C-T	R681C	SAOUHSC_00006 (*gyrA*)	DNA gyrase subunit A
	176626		G-A	D784N	SAOUHSC_00162 (*hsdR*)	Type I site-specific deoxyribonuclease
**TI3 (SJF4992)**	9045		C-T	R681C	SAOUHSC_00006 (*gyrA*)	DNA gyrase subunit A
	176626	4	G-A	D784N	SAOUHSC_00162 (*hsdR*)	Type I site-specific deoxyribonuclease
	2471861		C-T	H173Y	SAOUHSC_02685 (*nirR*)	Hypothetical protein
**Parental strain pRB474-p*mecA*-TI1 (SJF4984)**						
**TIR1 (SJF4986)**	9045		C-T	R681C	SAOUHSC_00006 (*gyrA*)	DNA gyrase subunit A
	176626	≥256	G-A	D784N	SAOUHSC_00162 (*hsdR*)	Type I site-specific deoxyribonuclease
	1718656		G-A	G54D	SAOUHSC_01812 (*pde2*)	DHH family phosphodiesterase
**TIR2 (SJF4987)**	9045		C-T	R681C	SAOUHSC_00006 (*gyrA*)	DNA gyrase subunit A
	18904	≥256	C-G	S196W	SAOUHSC_00015 (*gdpP*)	c-di-AMP phosphodiesterase
	176626		G-A	D784N	SAOUHSC_00162 (*hsdR*)	Type I site-specific deoxyribonuclease
**TIR3 (SJF4988)**	9045		C-T	R681C	SAOUHSC_00006 (*gyrA*)	DNA gyrase subunit A
	19903	≥256	T-C	F529S	SAOUHSC_00015 (*gdpP*)	c-di-AMP phosphodiesterase
	176626		G-A	D784N	SAOUHSC_00162 (*hsdR*)	Type I site-specific deoxyribonuclease
**Parental strain pRB474-p*mecA* (SJF4981)**						
**TR1 (SJF4985)**	19852	≥256	C-A	A512E	SAOUHSC_00015 (*gdpP*)	c-di-AMP phosphodiesterase
**TR2 (SJF4990)**	19188	≥256	G-C	G291R	SAOUHSC_00015 (*gdpP*)	c-di-AMP phosphodiesterase
**TR3 (SJF4991)**	19270		G-T	R318L	SAOUHSC_00015 (*gdpP*)	c-di-AMP phosphodiesterase
	376633	≥256	G-T	E212*	SAOUHSC_00370	Hypothetical protein
	1089537		C-G	P216A	SAOUHSC_01137	Hypothetical protein

S4 Table: Mutations identified by whole genome sequencing in SH1000 pRB474-p*mecA* derivatives relative to NCTC8325.
