# Supplementary material for: Evolving MRSA: High-level β-lactam resistance in Staphylococcus aureus is associated with RNA Polymerase alterations and fine tuning of gene expression
Source: PLoS Pathog. 2020 Jul 24;16(7):e1008672. doi: 10.1371/journal.ppat.1008672 (PMC7380596; doi:10.1371/journal.ppat.1008672)
Supplement: S5 Table — Frequencies of rifampicin-resistant colonies for strains carrying rpoBC mutations were determined from plates spread with 109 to 1011 viable cells supplemented with 5 and 100 μg/ml rifampicin. Frequency of RMPR mutants expressed as average (±SD) number of RMPR colonies from three independent experiments. <5×10^−11^ corresponds to no colonies detected. (PDF) [file ppat.1008672.s005.pdf]

Strains	Amino acid change	Oxacillin MIC (μg/ml)	RMP^R mutants	
			Rifampicin 5 μg/ml	Rifampicin 100 μg/ml
SJF682			$4.5 \pm 2.06 \times 10^{-8}$	$1.10 \pm 0.80 \times 10^{-8}$
SJF4996			$3.34 \pm 0.45 \times 10^{-8}$	$1.03 \pm 0.58 \times 10^{-8}$
SJF5003	*rpoB*-H929Q	≥256	$1.86 \pm 1.07 \times 10^{-9}$	$1.72 \pm 1.25 \times 10^{-9}$
SJF5034	*rpoC*-G740R	≥256	$5.23 \pm 2.66 \times 10^{-10}$	$1.76 \pm 0.82 \times 10^{-10}$
SJF4999	*rpoB*-G1139D	16	$1.20 \pm 0.10 \times 10^{-9}$	**$<5 \times 10^{-11}$**
SJF5008	*rpoB*-G639C, D949H	≥256	$4.07 \pm 2.7 \times 10^{-10}$	$1.43 \pm 0.06 \times 10^{-10}$
SJF5031	*rpoB*-Q643P	≥256	$1.01 \pm 1.85 \times 10^{-10}$	**$<5 \times 10^{-11}$**
SJF5000	*rpoC*-R739S	≥256	$5.67 \pm 2.31 \times 10^{-8}$	$3.57 \pm 1.10 \times 10^{-8}$
SJF5001	*rpoC*-I1084F	16	$3.90 \pm 1.64 \times 10^{-9}$	$3.00 \pm 1.80 \times 10^{-9}$
SJF5002	*rpoC*-S852Y	16	$4.03 \pm 0.91 \times 10^{-9}$	$1.18 \pm 0.37 \times 10^{-9}$
SJF5005	*rpoC*-E933Q	≥256	$2.97 \pm 0.95 \times 10^{-9}$	$2.4 \pm 0.45 \times 10^{-9}$
SJF5006	*rpoC*-A738T	≥256	$1.01 \pm 0.18 \times 10^{-8}$	$1.53 \pm 0.68 \times 10^{-8}$
SJF5007	*rpoC*-G950R	≥256	$2.00 \pm 0.30 \times 10^{-9}$	$2.10 \pm 1.15 \times 10^{-9}$

S5 Table: Mutations frequencies for rifampicin resistance in *S. aureus* strains.
Frequencies of rifampicin-resistant colonies for strains carrying *rpoBC* mutations were determined from plates spread with 10^9 to 10^{11} viable cells supplemented with 5 and 100 μg/ml rifampicin. Frequency of RMP^R mutants expressed as average (±SD) number of RMP^R colonies from three independent experiments. $<5 \times 10^{-11}$ corresponds to no colonies detected.
