# Supplementary material for: Evolving MRSA: High-level β-lactam resistance in Staphylococcus aureus is associated with RNA Polymerase alterations and fine tuning of gene expression
Source: PLoS Pathog. 2020 Jul 24;16(7):e1008672. doi: 10.1371/journal.ppat.1008672 (PMC7380596; doi:10.1371/journal.ppat.1008672)
Supplement: S6 Table — *, compared against WT; †, compared against lysA::pmecA (SJF4996); 26 DEGs shared by all three rpo mutant strains are highlighted in blue, related to Fig 6D. Clusters of orthologues groups of proteins (COGs) were retrieved from NCBI for 121 common DEGs associated with high-level resistance where, CELLULAR PROCESSES AND SIGNALING includes, [M] Cell wall/membrane/envelope biogenesis; [O] Post-translational modification, protein turnover, and chaperones; [T] Signal transduction mechanisms; [V] Defence mechanisms; INFORMATION STORAGE AND PROCESSING includes [J] Translation, ribosomal structure and biogenesis; [K] Transcription; [L] Replication, recombination and repair; METABOLISM includes [C] Energy production and conversion; [E] Amino acid transport and metabolism; [F] Nucleotide transport and metabolism; [G] Carbohydrate transport and metabolism; [H] Coenzyme transport and metabolism; [I] Lipid transport and metabolism; [P] Inorganic ion transport and metabolism; [Q] Secondary metabolites biosynthesis, transport, and catabolism; and POORLY CHARACTERIZED includes [R] General function prediction only; [S] Function unknown. (PDF) [file ppat.1008672.s006.pdf]

COG Functional categories	UniProt accession	Gene name	Locus tag	Protein product	Log2FoldChange				Predicated Regulators
					lysA::*pmecA* (SJF4996)*	*lysA*::*pmecA rpoB*-H929Q (SJF5003)†	*lysA*::*kan rpoB*-H929Q (SJF5010)†	*lysA*::*pmecA rpoC*-G740R (SJF5034)†	
**Cellular processes and signalling**									
VW	Q2G012	*emp*	SAOUHSC_00816	Extracellular matrix protein-binding protein emp	-1.77	1.88	-	1.41	-
V	Q2G140	*mepA*	SAOUHSC_00315	Multidrug export protein MepA (Staphylococcal virulence regulator protein A)	1.14	-1.06	-	-1.10	MepR
T	Q2FVM6	*nreB*	SAOUHSC_02676	Oxygen sensor histidine kinase NreB (Nitrogen regulation protein B)	1.12	-1.14	-	-1.29	Rex, NreC
O	Q2FZZ3	*yusE*	SAOUHSC_00841	Uncharacterized protein	-	1.33	-	1.82	SigB
VW	Q2FZB8	*fib*	SAOUHSC_01114	Fibrinogen-binding protein	-1.60	1.85	-	1.89	SaeR
V	Q2FYW3	*yvfR*	SAOUHSC_01311	ABC transporter, ATP-binding protein, putative	-	1.25	-	2.22	-
T	Q2FXL6	*ydaA/uspA1*	SAOUHSC_01819	Putative universal stress protein	-1.50	1.33	-	1.23	-
O	Q2FXE9	*spdA*	SAOUHSC_01900	Uncharacterized protein membrane spanning protein	-1.25	1.41	-	2.12	-
V	Q2FWP0	*lukG*	SAOUHSC_02241	Uncharacterized leukocidin-like protein 1	1.20	-1.17	-	-1.36	SaeR
M	Q2FW95	*fmtB*	SAOUHSC_02404	Extracellular matrix-binding protein	-1.28	1.19	-	3.04	-
M	Q2FVL5	*fmhA/femA*	SAOUHSC_02696	methicillin resistance determinant protein	-1.15	1.19	-	2.19	-
M	Q2FVH5		SAOUHSC_02737	Epimerase/dehydratase, putative	-1.35	1.22	-	1.20	-
V	Q2FVB4	*ydbJ*	SAOUHSC_02820	ABC-type multidrug transport system	-2.65	2.48	-	2.18	-
M	Q2FVB3		SAOUHSC_02821	Membrane spanning protein, putative	-2.09	1.97	-	1.60	-
O	Q2FV35		SAOUHSC_02904	Uncharacterized protein monooxygenase/thioredoxin reductase	-	-1.00	-	-1.05	Zur
V	Q2FUY3	*estA/xynC*	SAOUHSC_02962	Tributyrin esterase, putative	-1.69	1.22	-	2.23	-
O	Q2FUX4	*aur*	SAOUHSC_02971	Aureolysin, putative	-1.75	1.52	-	3.18	CodY
VM	Q2G2B2	*sasG*	SAOUHSC_02798	Surface protein G	-1.54	1.34	-	1.58	-
O	P72360	*scdA*	SAOUHSC_00229	Iron-sulfur cluster repair protein ScdA (Cell wall-related protein ScdA)	-1.50	1.33	-	1.17	-
DM	A0A0H2WXF8	*mecA*	SACOL0033	Penicillin-binding protein 2A	11.31	1.12	-11.19	0.60	-
O	Q2G1D7	*pflA*	SAOUHSC_00188	Pyruvate formate-lyase-activating enzyme	3.16	-3.72	-3.15	-4.10	CcpA, Rex
V	Q2FVK5	*sbi*	SAOUHSC_02706	Immunoglobulin-binding protein sbi	-1.63	1.66	1.40	1.38	SaeR
V	P02976	*spa*	SAOUHSC_00069	Immunoglobulin G-binding protein A (IgG-binding protein A)	-2.21	1.67	1.90	1.01	CcpA
**Cellular processes and signalling, information storage and processing**									
TK	Q2FVM7	*nreC*	SAOUHSC_02675	Oxygen regulatory protein NreC (Nitrogen regulation protein C)	1.31	-1.27	-	-1.20	Rex, NreC
**Information storage and processing**									
J	Q2FUQ2	*mnmE/trmE*	SAOUHSC_03053	tRNA modification GTPase MnmE	-1.68	1.33	-	1.96	-
L	O50581	*recG*	SAOUHSC_01194	ATP-dependent DNA helicase RecG	-1.58	1.59	-	2.30	-
L	Q2FZD7	*rnhC*	SAOUHSC_01095	Ribonuclease HIII (RNase HIII)	-1.12	1.00	-	1.18	-
K	Q2FUQ1	*rnpA*	SAOUHSC_03054	Ribonuclease P protein component (RNase P protein)	-2.10	1.67	-	2.53	-

L	Q2G057	*comFA*	SAOUHSC_00765	Uncharacterized protein comF operon protein 1	-1.68	1.49	-	2.65	-
J	Q2FZZ4	*yusF*	SAOUHSC_00840	5S rRNA maturation endonuclease (Ribonuclease M5)	-2.17	1.92	-	2.75	SigB
L	Q2FYS2	*uvrX*	SAOUHSC_01363	Nucleotidyltransferase	-1.21	1.26	-	2.08	LexA
L	Q2G2Y4		SAOUHSC_01918	Uncharacterized protein excalibur calcium-binding domain protein	1.04	-1.08	-	-1.58	-
L	Q2FWL3	*mutS*	SAOUHSC_02276	MutS domain V protein	-	1.12	-	1.27	-
K	Q2G0D1	*sarX*	SAOUHSC_00674	HTH-type transcriptional regulator SarX (Staphylococcal accessory regulator X)	1.54	-1.63	-	-3.24	-
L	Q2FZD0	*uvrC*	SAOUHSC_01102	UvrABC system protein C (Protein UvrC) (Excinuclease ABC subunit C)	-1.47	1.29	-	2.40	-
K	Q2G1V2	*nirR*	SAOUHSC_02685	Transcriptional regulator, Nitrite reductase	2.41	-3.45	-3.26	-3.70	Rex, NreC
**Information storage and processing or cellular processes and signalling**									
JU	Q2FYF4	*ansA*	SAOUHSC_01497	L-asparaginase, putative	-1.17	1.15	-	1.00	-
TK	Q2FWH6	*kdpE*	SAOUHSC_02315	DNA-binding response regulator, putative	-1.25	1.21	-	3.55	-
**Metabolism**									
E	Q2FZU1	*argG*	SAOUHSC_00899	Argininosuccinate synthase	1.11	-1.28	-	-1.36	ArgR, CodY
G	Q2FV87	*glcB*	SAOUHSC_02848	Phosphotransferase system IIC components, glucose/maltose/N-acetylglucosamine-specific	-	-1.11	-	-1.19	-
F	Q2G0Y6	*guaA*	SAOUHSC_00375	GMP synthase, PP-ATPase domain/subunit	1.13	-1.08	-	-1.18	-
E	Q2FUT6	*hisZ*	SAOUHSC_03015	ATP phosphoribosyltransferase regulatory subunit	1.00	-1.27	-	-1.06	CodY, HisR
I	Q2G155	*geh/lip2*	SAOUHSC_00300	Lipase 2 (Glycerol ester hydrolase 2)	-1.71	1.61	-	1.34	-
E	Q2G0V2	*metN1*	SAOUHSC_00423	Methionine import ATP-binding protein MetN 1	-1.21	1.06	-	1.39	CymR
F	Q2FZ75	*pyrB*	SAOUHSC_01166	Aspartate carbamoyltransferase	1.03	-1.13	-	-1.18	-
F	Q2FZ71	*pyrF*	SAOUHSC_01171	Orotidine 5'-phosphate decarboxylase (OMP decarboxylase)	1.18	-1.34	-	-1.57	-
F	Q2FZ77	*pyrR*	SAOUHSC_01164	Pyrimidine operon attenuation protein/uracil phosphoribosyltransferase	2.03	-2.12	-	-2.50	-
G	Q2G252	*rlmH*	SAOUHSC_00027	Ribosomal RNA large subunit methyltransferase H	-1.69	1.55	-	1.92	-
E	Q2G1H3	*rocD/argD*	SAOUHSC_00150	Acetylornithine/succinyldiaminopimelate/putrescine aminotransferase	-1.32	1.05	-	1.22	ArgR
F	Q2G253	*adsA*	SAOUHSC_00025	Uncharacterized protein	-2.94	2.60	-	2.41	-
P	Q2G1N5	*sirB*	SAOUHSC_00072	Lipoprotein, SirB, putative	-1.38	1.17	-	2.18	Fur
P	Q2G1N4	*sirA*	SAOUHSC_00074	Periplasmic binding protein, putative SirA	-1.59	1.50	-	1.99	Fur
E	Q2G1N2	*ocd2*	SAOUHSC_00076	Ornithine cyclodeaminase, putative	-1.16	1.03	-	2.53	Fur
G	Q2G145	*ulaA*	SAOUHSC_00310	PTS system ascorbate-specific transporter subunit IIC	-1.31	1.24	-	1.55	-
F	Q2G0Y8	*pbuX*	SAOUHSC_00373	Xanthine permease, putative	1.31	-1.26	-	-1.20	-
G	Q2G0U0	*treP*	SAOUHSC_00437	Uncharacterized protein	1.43	-1.35	-	-1.15	CcpA
H	Q2G0Q7	*folP*	SAOUHSC_00489	Dihydropteroate synthase (DHPS)	-1.55	1.55	-	1.81	-
H	Q2G0Q6	*folB*	SAOUHSC_00490	7,8-dihydroneopterin aldolase	-1.04	1.11	-	1.41	-
C	Q2G0M2		SAOUHSC_00538	Haloacid dehalogenase-like hydrolase, putative	-1.08	1.10	-	1.13	-
GEPR	Q2G0K4	*proP*	SAOUHSC_00556	Proline/betaine transporter, putative	1.63	-1.37	-	-1.22	SigB
I	Q2G0E5	*aes*	SAOUHSC_00661	Acetyl esterase/lipase	1.34	-1.36	-	-1.08	-
G	Q2G239	*fruA*	SAOUHSC_00708	Fructose specific permease, putative	1.23	-1.12	-	-1.22	FruR, CcpA

E	Q2FZQ1	*yrbD*	SAOUHSC_00949	Uncharacterized protein	-1.27	1.07	-	1.61	CodY
F	Q2FZ76	*pyrP*	SAOUHSC_01165	Uracil permease, putative	1.52	-1.44	-	-1.57	-
I	Q2FYZ3	*pldB*	SAOUHSC_01279	Hydrolase, alpha/beta fold family domain protein	-1.07	1.26	-	1.60	-
Q	Q2FY61		SAOUHSC_01604	Uncharacterized protein glyoxalase/bleomycin resistance protein/dioxygenase	-1.65	1.57	-	1.69	-
C	Q2FY54	*bfmBB*	SAOUHSC_01611	Dihydrolipoamide acetyltransferase component of pyruvate dehydrogenase complex	1.09	-1.03	-	-1.23	-
Q	Q2FXE2	*yvgN*	SAOUHSC_01907	Aldo/keto reductase, related to diketogulonate reductase	-1.64	1.35	-	1.81	-
E	Q2FV98	*yncA*	SAOUHSC_02836	L-amino acid N-acyltransferase	1.02	-1.18	-	-1.16	-
E	Q2G1N3	*sbnA*	SAOUHSC_00075	Probable siderophore biosynthesis protein SbnA	-1.18	1.04	-	2.62	Fur
P	Q2G261	*sodM*	SAOUHSC_00093	Superoxide dismutase [Mn/Fe]	-1.28	1.09	-	1.10	CodY
E	Q2FZL2	*sspA*	SAOUHSC_00988	V8-like Glu-specific endopeptidase	-1.36	1.07	-	1.68	-
E	Q2FZL3	*sspB*	SAOUHSC_00987	Staphylococcal cysteine proteinase B	-1.19	1.03	-	1.53	-
H	Q2FWG0	*tenA*	SAOUHSC_02331	Aminopyrimidine aminohydrolase (Thiaminase II)	-	1.16	-	1.73	-
E	Q2FVW5	*ureA*	SAOUHSC_02558	Urease subunit gamma	1.96	-1.88	-	-1.54	-
E	Q2G2K6	*ureB*	SAOUHSC_02559	Urease subunit beta	1.62	-1.38	-	-1.27	-
E	Q2G2K5	*ureC*	SAOUHSC_02561	Urease subunit alpha	1.72	-1.63	-	-1.32	-
F	Q2G0Y9	*xpt*	SAOUHSC_00372	Xanthine phosphoribosyltransferase (XPRTase)	1.09	-1.13	-	-1.00	-
H	Q2FVM0	*nasF/cobA*	SAOUHSC_02682	Uroporphyrin-III C-methyltransferase, putative	3.13	-3.33	-2.74	-3.49	Rex, NreC
C	Q2FVQ4	*lctP*	SAOUHSC_02648	L-lactate permease	1.85	-2.25	-1.72	-2.64	Rex
CP	Q2FVM2	*narH*	SAOUHSC_02680	Nitrate reductase, beta subunit	3.08	-3.49	-3.27	-3.74	Rex, NreC
C	Q2FVL8	*nirB/nasD*	SAOUHSC_02684	Assimilatory nitrite reductase [NAD(P)H], large subunit, putative	2.88	-3.40	-3.40	-3.78	Rex, NreC
CP	Q2FVM1	*narG*	SAOUHSC_02681	Nitrate reductase, alpha subunit	2.87	-3.73	-3.67	-4.06	Rex, NreC
C	Q2G218	*ldh1*	SAOUHSC_00206	L-lactate dehydrogenase 1 (L-LDH 1)	2.39	-3.45	-3.40	-4.13	Rex
C	Q2G1D8	*pflB*	SAOUHSC_00187	Formate acetyltransferase (Pyruvate formate-lyase)	2.87	-3.41	-3.05	-3.57	Rex, CcpA
P	Q2FVN1	*narT/narK*	SAOUHSC_02671	Probable nitrate transporter NarT	2.39	-2.94	-3.06	-2.96	Rex, NreC
P	Q2G172		SAOUHSC_00281	Uncharacterized protein, formate-nitrite transporter	2.21	-2.66	-2.40	-2.23	Rex
F	Q2FV02	*nrdD*	SAOUHSC_02942	Anaerobic ribonucleoside-triphosphate reductase, putative	1.51	-1.58	-1.46	-1.67	NrdR
E	Q2FYJ3	*tdcB*	SAOUHSC_01451	L-threonine dehydratase (Threonine deaminase)	2.37	-2.72	-3.11	-2.92	CodY
CPO	Q2FVM3	*narJ*	SAOUHSC_02679	Respiratory nitrate reductase, delta subunit, putative	2.63	-2.53	-3.03	-2.97	Rex, NreC
E	Q2FYJ4		SAOUHSC_01450	Uncharacterized protein amino acid permease	1.50	-1.76	-2.06	-2.17	CodY
G	Q2G0G1	*adh*	SAOUHSC_00608	Alcohol dehydrogenase	1.97	-2.15	-2.34	-2.57	Rex
C	Q2FYJ2	*ald1*	SAOUHSC_01452	Alanine dehydrogenase 1	1.84	-2.31	-2.50	-2.52	Rex
PQ	Q2FVL9	*nirD/nasE*	SAOUHSC_02683	Assimilatory nitrite reductase [NAD(P)H], small subunit, putative	2.07	-2.38	-2.40	-2.41	Rex, NreC
C	Q2G1K9	*adhE*	SAOUHSC_00113	Aldehyde-alcohol dehydrogenase	1.63	-1.85	-1.78	-1.80	Rex
**Metabolism and cellular processes and signalling**									
CO	Q2G0B2	*cydC*	SAOUHSC_00693	ATP-binding/permease protein	1.12	-1.10	-	-1.23	-
GM	Q9RQP7	*icaB*	SAOUHSC_03004	Peptidoglycan/xylan/chitin deacetylase, PgdA/CDA1 family	-1.33	1.06	-1.08	-1.04	CodY, IcaR

**Poorly characterised**									
R	Q2FZC0	*flr*	SAOUHSC_01112	FPRL1 inhibitory protein	-1.35	1.86	-	1.93	-
S	Q2G249		SAOUHSC_00026	Uncharacterized protein	-1.53	1.37	-	1.56	-
S	Q2G1N1	*sbnC*	SAOUHSC_00077	Uncharacterized protein	-1.02	1.00	-	2.41	Fur
S	Q2G177		SAOUHSC_00270	Uncharacterized protein putative lipoprotein	-	1.32	-	1.37	-
S	Q2G176		SAOUHSC_00271	Uncharacterized protein	-	1.14	-	1.27	-
S	Q2G105		SAOUHSC_00356	Uncharacterized protein	-1.65	1.88	-	1.22	SigB
S	Q2G0Y5		SAOUHSC_00376	Uncharacterized protein	-1.07	1.53	-	2.23	-
S	Q2G0X2		SAOUHSC_00401	Uncharacterized protein	-1.74	1.94	-	1.90	-
S	Q2G0E3		SAOUHSC_00662	Uncharacterized protein	1.26	-1.13	-	-1.25	-
S	Q2G2G0	*SaeP*	SAOUHSC_00717	Uncharacterized protein putative lipoprotein	-1.16	1.41	-	1.46	SaeR
R	Q2G035	*yfcH*	SAOUHSC_00792	Epimerase family protein	-2.45	2.12	-	2.62	SigB
R	Q2G016	*yjhQ/yhbs*	SAOUHSC_00811	Predicted N-acetyltransferase	-1.96	1.80	-	2.22	SigB
S	Q2FZZ6		SAOUHSC_00838	Uncharacterized protein	-3.62	3.18	-	4.14	-
S	Q2FZT3		SAOUHSC_00907	UPF0344 membrane protein	-	1.00	-	1.22	-
R	Q2G1U4	*trfB*	SAOUHSC_00936	Uncharacterized protein, transcription factor	-	1.01	-	2.36	-
S	Q2G200		SAOUHSC_00941	UPF0738 protein	-1.54	1.51	-	1.37	-
S	Q2FZB9		SAOUHSC_01113	Uncharacterized protein membrane protein	-	1.35	-	1.34	-
S	Q2FZ03		SAOUHSC_01268	Uncharacterized protein	-1.08	1.32	-	1.80	-
S	Q2FXQ3	*ymaB*	SAOUHSC_01782	Uncharacterized protein	-1.38	1.10	-	2.10	-
S	Q2G2Y3		SAOUHSC_01919	Uncharacterized protein membrane protein	1.13	-1.13	-	-1.53	-
R	Q2FV53		SAOUHSC_02886	Uncharacterized protein	-	1.13	-	1.97	-
S	Q2G1H7		SAOUHSC_00146	Uncharacterized protein integral membrane protein	-1.42	-2.01	-1.42	-2.53	-
R	Q2FV03	*nrdG*	SAOUHSC_02941	Anaerobic ribonucleoside-triphosphate reductase-activating protein	1.62	-1.61	-1.60	-1.75	NrdR
R	Q2FZC2	*ecb*	SAOUHSC_01110	Fibrinogen-binding protein-related	-1.16	1.33	1.17	1.29	SaeR

S6 Table: Functional classification and differential gene expression of shared gene pools of *mecA*⁺ and *mecA*⁺ *rpo* strains.
*, compared against WT; †, compared against *lysA*::p*mecA* (SJF4996); 26 DEGs shared by all three *rpo* mutant strains are highlighted in blue, related to Figure 6D. Clusters of orthologues groups of proteins (COGs) were retrieved from NCBI for 121 common DEGs associated with high-level resistance where, CELLULAR PROCESSES AND SIGNALING includes, [M] Cell wall/membrane/envelope biogenesis; [O] Post-translational modification, protein turnover, and chaperones; [T] Signal transduction mechanisms; [V] Defence mechanisms; INFORMATION STORAGE AND PROCESSING includes [J] Translation, ribosomal structure and biogenesis; [K] Transcription; [L] Replication, recombination and repair; METABOLISM includes [C] Energy production and conversion; [E] Amino acid transport and metabolism; [F] Nucleotide transport and metabolism; [G] Carbohydrate transport and metabolism; [H] Coenzyme transport and metabolism; [I] Lipid transport and metabolism; [P] Inorganic ion transport and metabolism; [Q] Secondary metabolites biosynthesis, transport, and catabolism; and POORLY CHARACTERIZED includes [R] General function prediction only; [S] Function unknown.
