# Supplementary material for: Evolving MRSA: High-level β-lactam resistance in Staphylococcus aureus is associated with RNA Polymerase alterations and fine tuning of gene expression
Source: PLoS Pathog. 2020 Jul 24;16(7):e1008672. doi: 10.1371/journal.ppat.1008672 (PMC7380596; doi:10.1371/journal.ppat.1008672)
Supplement: S7 Table — *, denotes trained strains with intermediate oxacillin resistance (TI); †, denotes trained strains with high-level oxacillin resistance (TR); ‡, denotes TI strain trained further for high-level oxacillin resistance (TIR). (PDF) [file ppat.1008672.s007.pdf]

Strain	Relevant genotypes/characteristics	Source
S. aureus		
SH1000	Functional *rsbU*+ derivative of 8325-4; (SJF682).	[1]
RN4220	Restriction deficient transformation recipient.	[2]
SJF4981	pRB474-p*mecA.* SH1000 carrying plasmid-borne *mecA* (pRB474-p*mecA*); Cm^R.	This study
SJF4984	SJF4981 derived strain TI1*, pRB474-p*mecA.* Cm^R.	This study
SJF4989	SJF4981 derived strain TI2*, pRB474-p*mecA.* Cm^R.	This study
SJF4992	SJF4981 derived strain TI3*, pRB474-p*mecA.* Cm^R.	This study
SJF5194	SJF4981 derived strain TI11*, pRB474-p*mecA.* Cm^R.	This study
SJF4985	SJF4981 derived strain TR1†, pRB474-p*mecA gdpP*-A512E. Cm^R.	This study
SJF4990	SJF4981 derived strain TR2†, pRB474-p*mecA gdpP*-G291R. Cm^R.	This study
SJF4991	SJF4981 derived strain TR3†, pRB474-p*mecA gdpP*-R318L. Cm^R.	This study
SJF4986	SJF4984 derived strain TIR1‡, pRB474-p*mecA pde2*-G54D. Cm^R.	This study
SJF4987	SJF4984 derived strain TIR2‡, pRB474-p*mecA gdpP*-S196W. Cm^R.	This study
SJF4988	SJF4984 derived strain TIR3‡, pRB474-p*mecA gdpP*-F529S. Cm^R.	This study
SJF4993	*gdpP*-R318L; SJF4991 cured off pRB474-p*mecA*.	This study
SJF4995	*gdpP*-R318L pRB474-p*mecA*+; SJF4993 reintroduced with pRB474-p*mecA.* Cm^R.	This study
ANG1959	SEJ1 Δ*gdpP*::*Kan*^{R}; marked *gdpP* deletion.	[3]
SJF5025	SH1000 Δ*gdpP*::*Kan*^{R}.	This study
SJF5026	SJF5025 with Δ*gdpP*::*Kan*^{R}, pRB474-p*mecA,* carrying plasmid-borne *mecA.* Cm^R.	This study
SJF4994	RN4220 *lysA*::p*mecA.* Chromosomally integrated single copy *mecA* expressed via its native promoter at *lysA* locus. Ery^R, Lin^R.	This study
SJF4996	SH1000 with *lysA*::p*mecA,* carrying chromosomally integrated single copy *mecA* expressed via its native promoter at *lysA* locus. Ery^R, Lin^R.	This study
SJF4998	SJF4996 derived strain TI1*, *lysA*::p*mecA*. Ery^R, Lin^R.	This study
SJF4999	SJF4996 derived strain TI2*, *lysA*::p*mecA rpoB*-G1139D. Ery^R, Lin^R.	This study
SJF5001	SJF4996 derived strain TI3*, *lysA*::p*mecA rpoC*-I1084F*.* Ery^R, Lin^R.	This study
SJF5002	SJF4996 derived strain TI4*, *lysA*::p*mecA rpoC*-S852Y. Ery^R, Lin^R.	This study
SJF5000	SJF4996 derived strain TR1†, *lysA*::p*mecA rpoC*-R739S. Ery^R, Lin^R.	This study
SJF5003	SJF4996 derived strain TR2†, *lysA*::p*mecA rpoB*-H929Q. Ery^R, Lin^R.	This study
SJF5004	SJF4996 derived strain TR3†, *lysA*::p*mecA rpoB*-H929Q. Ery^R, Lin^R.	This study
SJF5005	SJF4996 derived strain TR4†, *lysA*::p*mecA rpoC*-E933Q. Ery^R, Lin^R.	This study
SJF5031	SJF4996 derived strain TR5†, *lysA*::p*mecA rpoB*-Q643P. Ery^R, Lin^R.	This study
SJF5032	SJF4996 derived strain TR6†, *lysA*::p*mecA rpoB*-Q643P. Ery^R, Lin^R.	This study
SJF5033	SJF4996 derived strain TR7†, *lysA*::p*mecA rpoB*-Q643P. Ery^R, Lin^R.	This study
SJF5034	SJF4996 derived strain TR8†, *lysA*::p*mecA rpoC*-G740R. Ery^R, Lin^R.	This study
SJF5006	SJF4998 derived strain TIR1‡, *lysA*::p*mecA rpoC*-A738T. Ery^R, Lin^R.	This study
SJF5007	SJF4998 derived strain TIR2‡, *lysA*::p*mecA rpoC*-G950R. Ery^R, Lin^R.	This study
SJF5008	SJF4998 derived strain TIR3‡, *lysA*::p*mecA rpoB*-G639C D949H. Ery^R, Lin^R.	This study
SJF5009	RN4220 *lysA::kan*; empty vector at *lysA.*	This study

SJF5010	*lysA*::*kan rpoB*-H929Q; SJF5003 allele *lysA*::p*mecA* replaced with *lysA*::*kan* to remove chromosomal *mecA*.	This study
SJF5011	*lysA*::p*mecA*$^{+}$ *rpoB*-H929Q; SJF5010 reintroduced with *lysA*::p*mecA.* Ery^{R}, Lin^{R}.	This study
SJF4997	*gdpP*-R318L *lysA*::p*mecA*; SJF4993 with *lysA*::p*mecA*. Ery^{R}, Lin^{R}.	This study
SJF5024	*lysA*::*kan rpoB*-H929Q pRB474-p*mecA*; SJF5010 carrying pRB474-p*mecA*. Cm^{R}.	This study
8325-4	Restriction deficient derivative of 8325.	[2]
SJF5035	8325-4 *lysA*::p*mecA*. Ery^{R}, Lin^{R}.	This study
COL	HA-MRSA (type I SCC*mec*).	[4]
MRSA252	HA-MRSA (type II SCC*mec*).	[5]
Mu50	HA-MRSA (VISA clinical isolate).	[6]
Mu3	HA-MRSA (hVISA clinical isolate).	[7]
USA300_FPR3757	CA-MRSA (type IV SCC*mec*).	[8]
MW2	CA-MRSA (type IV SCC*mec*).	[9]
AJ1008	AR1089, with *kanA* near *rpoBC*, Kan^{R}.	[10]
MV42	AR1089, *ermB* near *rpoB*+, Ery^{R}. erythromycin cassette inserted near *rpoB* genomic region.	[10]
SJF5044	*lysA*::p*mecA rpoB*$^{+}$, SJF5003 genetically complemented to carry wild-type *rpoB* allele with *kan* nearby.	This study
SJF5045	*lysA*::p*mecA rpoC*$^{+}$, SJF5034 genetically complemented to carry wild-type *rpoC* allele with *kan* nearby.	This study
SJF5049	COL *rpoB*$^{+}$, COL *rpoB* allele replaced with wild-type *rpoB* allele with *ermB* nearby.	This study
SJF5046	SJF5003, *lysA*::p*mecA rpoB*-H929Q *kan*; selection of *rpoB*-H929Q with *kan* nearby. Kan^{R}, Ery^{R}, Lin^{R}.	This study
Newman	Clinical isolate (ATCC25904), *rsbU*$^{+}$. High-level clumping factor.	[11]
SJF5048	Newman *rpoB*-H929Q *kan*, introduction of *rpoB*-H929Q with *kan* nearby.	This study
SJF5050	Newman *lysA*::p*mecA rpoB*-H929Q *kan*. Kan^{R}, Ery^{R}, Lin^{R}.	This study
SJF5319	RN4220 *geh*::p*mecA.* Chromosomally integrated single copy *mecA* expressed via its native promoter at *geh* locus. Kan^{R}.	This study
SJF5320	*lysA*::*tet rpoB*-H929Q; SJF5010 allele *lysA*::*kan* replaced with *lysA*::*tet* to allow kanamycin selection of *geh*::p*mecA* (SJF5323).	This study
SJF5323	SJF5320 derived oxacillin resistant strain; *geh*::p*mecA lysA*::*tet rpoB*-H929Q. Kan^{R}, Tet^{R}.	This study
SJF5324	SH1000 *geh*::p*mecA.* Chromosomally integrated single copy *mecA* expressed via its native promoter at *geh* locus. Tet^{R}.	This study
SJF5331	SJF5323 derived strain; *geh*::p*mecA*-*kan lysA*::*tet rpoB*-H929Q *SAOUHSC_00271*::Tn. Kan^{R}, Tet^{R}, Ery^{R}.	This study
SJF5332	SJF5323 derived strain; *geh*::p*mecA*-*kan lysA*::*tet rpoB*-H929Q *SAOUHSC_00841* (*yusE*)::Tn. Kan^{R}, Tet^{R}, Ery^{R}.	This study
SJF5333	SJF5323 derived strain; *geh*::p*mecA*-*kan lysA*::*tet rpoB*-H929Q *SAOUHSC_00907*::Tn. Kan^{R}, Tet^{R}, Ery^{R}.	This study
SJF5334	SJF5323 derived strain; *geh*::p*mecA*-*kan lysA*::*tet rpoB*-H929Q *SAOUHSC*_00936 (*trfB*)::Tn. Kan^{R}, Tet^{R}, Ery^{R}.	This study
SJF5335	SJF5323 derived strain; *geh*::p*mecA*-*kan lysA*::*tet rpoB*-H929Q *SAOUHSC_01113*::Tn. Kan^{R}, Tet^{R}, Ery^{R}.	This study
SJF5336	SJF5323 derived strain; *geh*::p*mecA*-*kan lysA*::*tet rpoB*-H929Q *SAOUHSC_01311* (*yvfR*)::Tn. Kan^{R}, Tet^{R}, Ery^{R}.	This study

SJF5337	SJF5323 derived strain; *geh::pmecA-kan lysA::tet rpoB*-H929Q *SAOUHSC_02276* (*mutS*)::Tn. Kan^{R}, Tet^{R}, Ery^{R}.	This study
SJF5338	SJF5323 derived strain; *geh*::p*mecA-kan lysA*::*tet rpoB*-H929Q *SAOUHSC_02331* (*tenA*)::Tn. Kan^{R}, Tet^{R}, Ery^{R}.	This study
SJF5339	SJF5323 derived strain; *geh*::p*mecA-kan lysA*::*tet rpoB*-H929Q *SAOUHSC_02886* (*glcB*)::Tn. Kan^{R}, Tet^{R}, Ery^{R}.	This study
SJF5340	SJF5323 derived strain; *geh*::p*mecA-kan lysA*::*tet rpoB*-H929Q *SAOUHSC_02273* (*rex*)::Tn. Kan^{R}, Tet^{R}, Ery^{R}.	This study
SJF5341	SJF5323 derived strain; *geh*::p*mecA-kan lysA*::*tet rpoB*-H929Q *SAOUHSC_00113* (*adhE*)::Tn. Kan^{R}, Tet^{R}, Ery^{R}.	This study
SJF5342	SJF5323 derived strain; *geh*::p*mecA-kan lysA*::*tet rpoB*-H929Q *SAOUHSC_01450*::Tn. Kan^{R}, Tet^{R}, Ery^{R}.	This study
SJF5343	SJF5323 derived strain; *geh*::p*mecA-kan lysA*::*tet rpoB*-H929Q *SAOUHSC_00187* (*pflB*)::Tn. Kan^{R}, Tet^{R}, Ery^{R}.	This study
SJF5353	SJF5323 derived strain; *geh*::p*mecA-kan lysA*::*tet rpoB*-H929Q *SAOUHSC_00270*::Tn. Kan^{R}, Tet^{R}, Ery^{R}.	This study
SJF5344	SJF5003 derived strain; *lysA::pmecA rpoB-H929Q ΔSAOUHSC_00271*. Ery^{R}.	This study
E. coli		
DH5α	*E. coli, fhuA2Δ(argF-lacZ)U169 phoA glnV44 Φ80Δ (lacZ)M15 gyrA96 recA1 relA1 endA1 thi-1 hsdR17.*	New England Biolabs
SJF4983	DH5α *E. coli* pVP01-p*mecA*; Amp^{R}.	This study
SJF5316	DH5α *E. coli* pVP02-p*mecA*; Amp^{R}.	This study
**Plasmid**		
pRB474	Low copy *E. coli-Staphylococcus* shuttle vector. Amp^{R} (*E. coli*), Cm^{R} (*S. aureus*).	[12]
p*mecA*	2,867 bp EcoRI fragment containing *mecA* from pSA*mecA*5 subcloned into the EcoRI site of pRB474.	[13,14]
pMUTIN4	Derivative of pMUTIN which contains promoterless transcriptional *lacZ* fusion, non-replicating in gram positive bacteria; Amp^{R} (*E. coli*), Ery^{R}, Lin^{R} (*S. aureus*).	[15]
pGM068	pMUTIN4 derived insertion vector including *lysA* 3' fragment.	[16]
pVP01-p*mecA*	pGM068*,* insertion vector carrying *mecA* under its native promoter; Amp^{R} (*E. coli*), Ery^{R}, Lin^{R} (*S. aureus*).	This study
pVP02-p*mecA*	pKASBAR, insertion vector carrying *mecA* under its native promoter; Amp^{R} (*E. coli*), Kan^{R} (*S. aureus*).	This study
pIMAY	pIMAY-Δ*SAOUHSC_00271*, Amp^{R} (*E. coli*).	Tracy Palmer

References:

1. Horsburgh MJ, Aish JL, White IJ, Shaw L, Lithgow JK, Foster SJ. σ^{B} Modulates Virulence Determinant Expression and Stress Resistance: Characterization of a Functional *rsbU* Strain Derived from *Staphylococcus aureus* 8325-4. J Bacteriol. 2002;184(19):5457–67.
2. Kreiswirth BN, Löfdahl S, Betley MJ, O'reilly M, Schlievert PM, Bergdoll MS, et al. The toxic shock syndrome exotoxin structural gene is not detectably transmitted by a prophage. Nature. 1983;305:709–12.
3. Corrigan RM, Abbott JC, Burhenne H, Kaever V, Gründling A. C-di-amp is a new second messenger in *Staphylococcus aureus* with a role in controlling cell size and envelope stress. PLoS Pathog. 2011;7(9).
4. Shafer WM, Iandolo JJ. Genetics of staphylococcal enterotoxin B in methicillin-resistant isolates of *Staphylococcus aureus*. InfectImmun. 1979;25(3):902–11.

5. Enright MC, Day NPJ, Davies CE, Peacock SJ, Spratt BG. Multilocus Sequence Typing for Characterization of Methicillin-Resistant and Methicillin-Susceptible Clones of *Staphylococcus aureus*. J Clin Microbiol. 2000;38(3):1008–15.
6. Hiramatsu K, Hanaki H, Ino T, Yabuta K, Oguri T, Tenover FC. Methicillin-resistant *Staphylococcus aureus* clinical strain with reduced vancomycin susceptibility. J Antimicrob Chemother. 1997;40(1):135–6.
7. Hiramatsu K, Aritaka N, Hanaki H, Kawasaki S, Hosoda Y, Hori S, et al. Dissemination in Japanese hospitals of strains of *Staphylococcus aureus* heterogeneously resistant to vancomycin. Lancet. 1997;350(9092):1670–3.
8. Fey PD, Endres JL, Yajjala K, Widhelm TJ, Boissy RJ, Bose JL, et al. A Genetic Resource for Rapid and Comprehensive Phenotype Screening of Nonessential *Staphylococcus aureus* Genes. MBio. 2013;4(1):e00537-12-e00537-12.
9. Baba T, Takeuchi F, Kuroda M, Yuzawa H, Aoki KI, Oguchi A, et al. Genome and virulence determinants of high virulence community-acquired MRSA. Lancet. 2002;359(9320):1819–27.
10. Villanueva M, Jousselin A, Baek KT, Prados J, Andrey DO, Renzoni A, et al. Rifampin Resistance rpoB Alleles or Multicopy Thioredoxin/Thioredoxin Reductase Suppresses the Lethality of Disruption of the Global Stress Regulator *spx* in *Staphylococcus aureus*. J Bacteriol. 2016;198(July):JB.00261-16.
11. Duthie ES, Lorenz LL. Staphylococcal Coagulase: Mode of Action and Antigenicity. Microbiology. 1952;6(1–2):95–107.
12. Brückner R. Gene replacement in *Staphylococcus carnosus* and *Staphylococcus xylosus*. FEMS Microbiol Lett. 1997;151(1):1–8.
13. Pozzi C, Waters EM, Rudkin JK, Schaeffer CR, Lohan AJ, Tong P, et al. Methicillin resistance alters the biofilm phenotype and attenuates virulence in *Staphylococcus aureus* device-associated infections. PLoS Pathog. 2012;8(4).
14. Rudkin JK, Edwards AM, Bowden MG, Brown EL, Pozzi C, Waters EM, et al. Methicillin resistance reduces the virulence of healthcare-associated methicillin-resistant *Staphylococcus aureus* by interfering with the *agr* quorum sensing system. J Infect Dis. 2012;205(5):798–806.
15. Vagner V, Dervyn E, Ehrlich SD. A vector for systematic gene inactivation in *Bacillus subtilis*. Microbiology. 1998;144(11):3097–104.
16. McVicker G, Prajsnar TK, Williams A, Wagner NL, Boots M, Renshaw S, et al. Clonal Expansion during *Staphylococcus aureus* Infection Dynamics Reveals the Effect of Antibiotic Intervention. PLoS Pathog. 2014;10(2).

S7 Table: List of strains and plasmids used in this study

*, denotes trained strains with intermediate oxacillin resistance (TI); †, denotes trained strains with high-level oxacillin resistance (TR); ‡, denotes TI strain trained further for high-level oxacillin resistance (TIR).
