# Supplementary material for: Evolving MRSA: High-level β-lactam resistance in Staphylococcus aureus is associated with RNA Polymerase alterations and fine tuning of gene expression
Source: PLoS Pathog. 2020 Jul 24;16(7):e1008672. doi: 10.1371/journal.ppat.1008672 (PMC7380596; doi:10.1371/journal.ppat.1008672)
Supplement: S8 Table — (PDF) [file ppat.1008672.s008.pdf]

Target gene	Primer names	Primers sequence (5'-3')	Source
**p*mecA***	*mecA*_F1	CCTGACGATTCCAATGACGAAC	This study
	*mecA*_R1	CTTATTCATCTATATCGTATTTTTTATTACCGTTC	This study
lysA	*lysA*_5'_F	ATGGCGAATTAACAATGGATG	This study
	*lysA*_3'_R	CAAGCAATGCTTGATACTTTGCGTC	This study
rpoB	RNAP_F1	GAATCTGTTTGGCAGGTCAAGTTG	This study
	RNAP_R2	GAAATTATTTACATCAATCAAGGA	This study
rpoC	RNAP_F2	GATTAATACGCAATTTACAAAAC	This study
	RNAP_R1	TCCTCCAAAGTTCTGCTTGCATC	This study
kan* nearby *rpoBC	Kpn_rpoC_nearby5	ATGCGGTACCCTTGTAACGCACGAC ATGGTG	[1]
	Pst1_rpoC_nearby	GCATCTGCAGGCATCACGACCACTG CGTTGTTC	[1]
gdpP	VP62	CTATTGGTTATCGGTACAATACTGAC	This study
	VP63	GTTGCTTCTACAGCATAATTCTTTTTC	This study

References:

1. Villanueva M, Jousselin A, Baek KT, Prados J, Andrey DO, Renzoni A, et al. Rifampin Resistance *rpoB* Alleles or Multicopy Thioredoxin/Thioredoxin Reductase Suppresses the Lethality of Disruption of the Global Stress Regulator spx in *Staphylococcus aureus*. J Bacteriol. 2016;198(July):JB.00261-16.

S8 Table: List of oligonucleotides used in this study.
