# Supplementary material for: Evolving MRSA: High-level β-lactam resistance in Staphylococcus aureus is associated with RNA Polymerase alterations and fine tuning of gene expression
Source: PLoS Pathog. 2020 Jul 24;16(7):e1008672. doi: 10.1371/journal.ppat.1008672 (PMC7380596; doi:10.1371/journal.ppat.1008672)
Supplement: S1 Fig — A) Oxacillin susceptibility for parental and genetically complemented strains (stated as above) were compared using the Etest method. Oxacillin MICs are listed in brackets. B) The amounts of PBP2A (~76kDa) was determined using whole cell lysates of lysA::pmecA rpoB-H929Q (SJF5003), lysA::pmecA rpoC-G740R (SJF5034) and genetically complemented lysA::pmecA rpoB+ (SJF5044) and lysA::pmecA rpoC+ (SJF5045) as well as COL and COL rpoB+ (SJF5049) strains. (PDF) [file ppat.1008672.s009.pdf]

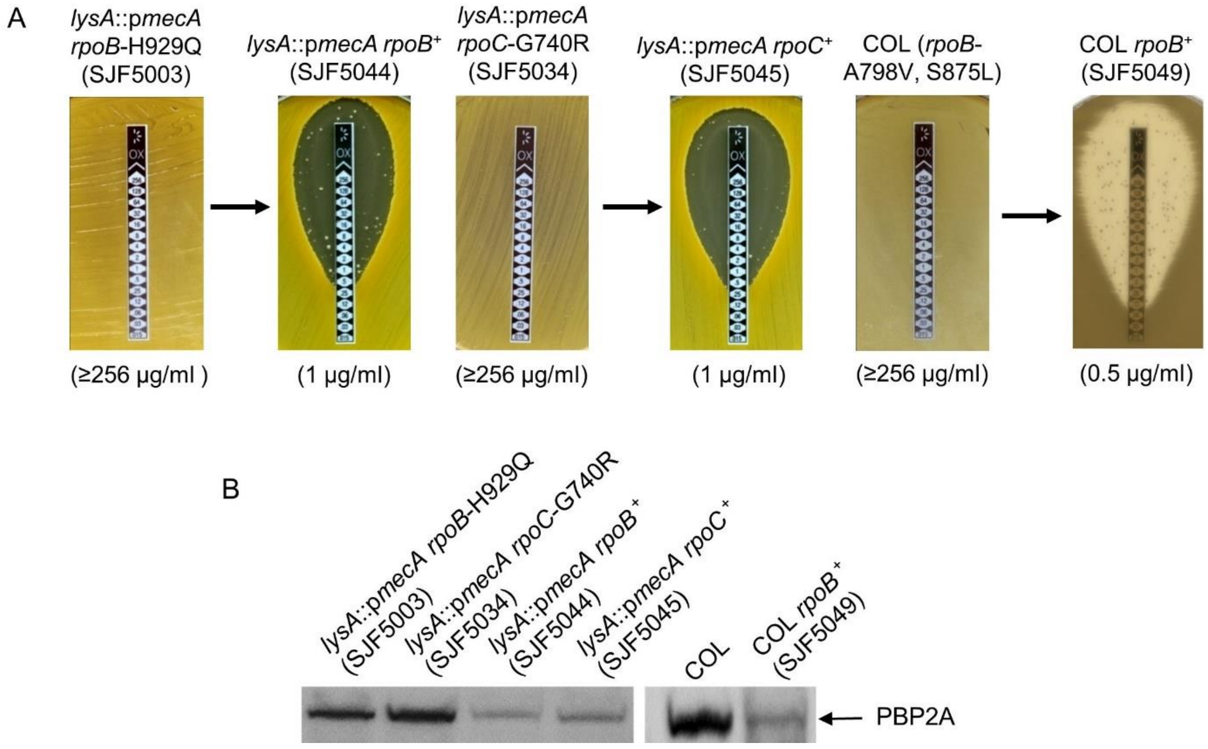

S1 Figure: Oxacillin resistance and levels of PBP2A in *rpoB/C* complemented strains. **A)** Oxacillin susceptibility for parental and genetically complemented strains (stated as above) were compared using the Etest method. Oxacillin MICs are listed in brackets. **B)** The amounts of PBP2A (~76kDa) was determined using whole cell lysates of *lysA*::p*mecA rpoB*-H929Q (SJF5003), *lysA*::p*mecA rpoC*-G740R (SJF5034) and genetically complemented *lysA*::p*mecA rpoB*$^{+}$ (SJF5044) and *lysA*::p*mecA rpoC*$^{+}$ (SJF5045) as well as COL and COL *rpoB*$^{+}$ (SJF5049) strains.
