# Supplementary material for: Evolving MRSA: High-level β-lactam resistance in Staphylococcus aureus is associated with RNA Polymerase alterations and fine tuning of gene expression
Source: PLoS Pathog. 2020 Jul 24;16(7):e1008672. doi: 10.1371/journal.ppat.1008672 (PMC7380596; doi:10.1371/journal.ppat.1008672)
Supplement: S2 Fig — A) RNA was radiolabelled on the 3’ end to produce an RNA of 14 nt. Nucleotide addition resulted in the production of an RNA product of 15 nt. (I) Schematic of scaffold before and after nucleotide addition. (II) 23% w/v polyacrylamide denaturing gel showing nucleotide addition over time. Observed rate constants (Kobs) are shown below the gel (numbers that follow the ± sign are standard errors). B) RNA of was radiolabelled on the 5’ end to produce an RNA of 13 nt. Pyrophosphorolysis first resulted in the production of an RNA product of 14 nt, subsequent pyrophosphorolysis produced shorter products. (I) Schematic of scaffold before and after pyrophosphorolysis. (II) 23% w/v polyacrylamide denaturing gel showing pyrophosphorolysis over time. Observed rate constants (Kobs) are shown below the gel (numbers that follow the ± sign are standard errors). (PDF) [file ppat.1008672.s010.pdf]

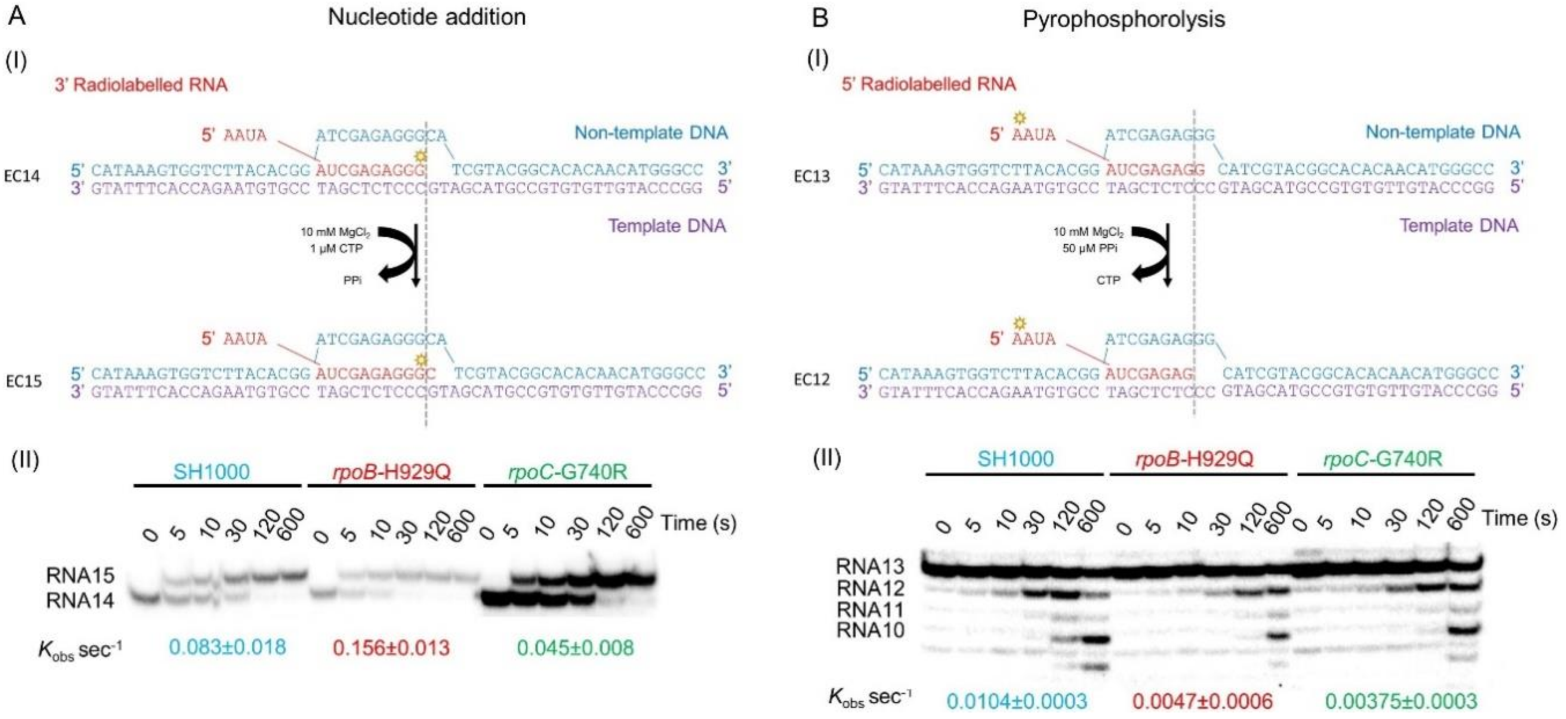

S2 Figure: Nucleotide addition and pyrophosphorolysis by SH1000, *lysA*::p*mecA rpoB*-H929Q and *lysA*::p*mecA rpoC*-G740R RNAPs. **A)** RNA was radiolabelled on the 3' end to produce an RNA of 14 nt. Nucleotide addition resulted in the production of an RNA product of 15 nt. (I) Schematic of scaffold before and after nucleotide addition. (II) 23% w/v polyacrylamide denaturing gel showing nucleotide addition over time. Observed rate constants (K_{obs}) are shown below the gel (numbers that follow the ± sign are standard errors). **B)** RNA of was radiolabelled on the 5' end to produce an RNA of 13 nt. Pyrophosphorolysis first resulted in the production of an RNA product of 14 nt, subsequent pyrophosphorolysis produced shorter products. (I) Schematic of scaffold before and after pyrophosphorolysis. (II) 23% w/v polyacrylamide denaturing gel showing pyrophosphorolysis over time. Observed rate constants (K_{obs}) are shown below the gel (numbers that follow the ± sign are standard errors).
