# Supplementary material for: Evolving MRSA: High-level β-lactam resistance in Staphylococcus aureus is associated with RNA Polymerase alterations and fine tuning of gene expression
Source: PLoS Pathog. 2020 Jul 24;16(7):e1008672. doi: 10.1371/journal.ppat.1008672 (PMC7380596; doi:10.1371/journal.ppat.1008672)
Supplement: S3 Fig — The assembled scaffold was used to examine differences in pausing by three RNAPs during elongation. The trace of each reaction at 30 seconds on the 23% w/v polyacrylamide denaturing gels can be seen. (PDF) [file ppat.1008672.s011.pdf]

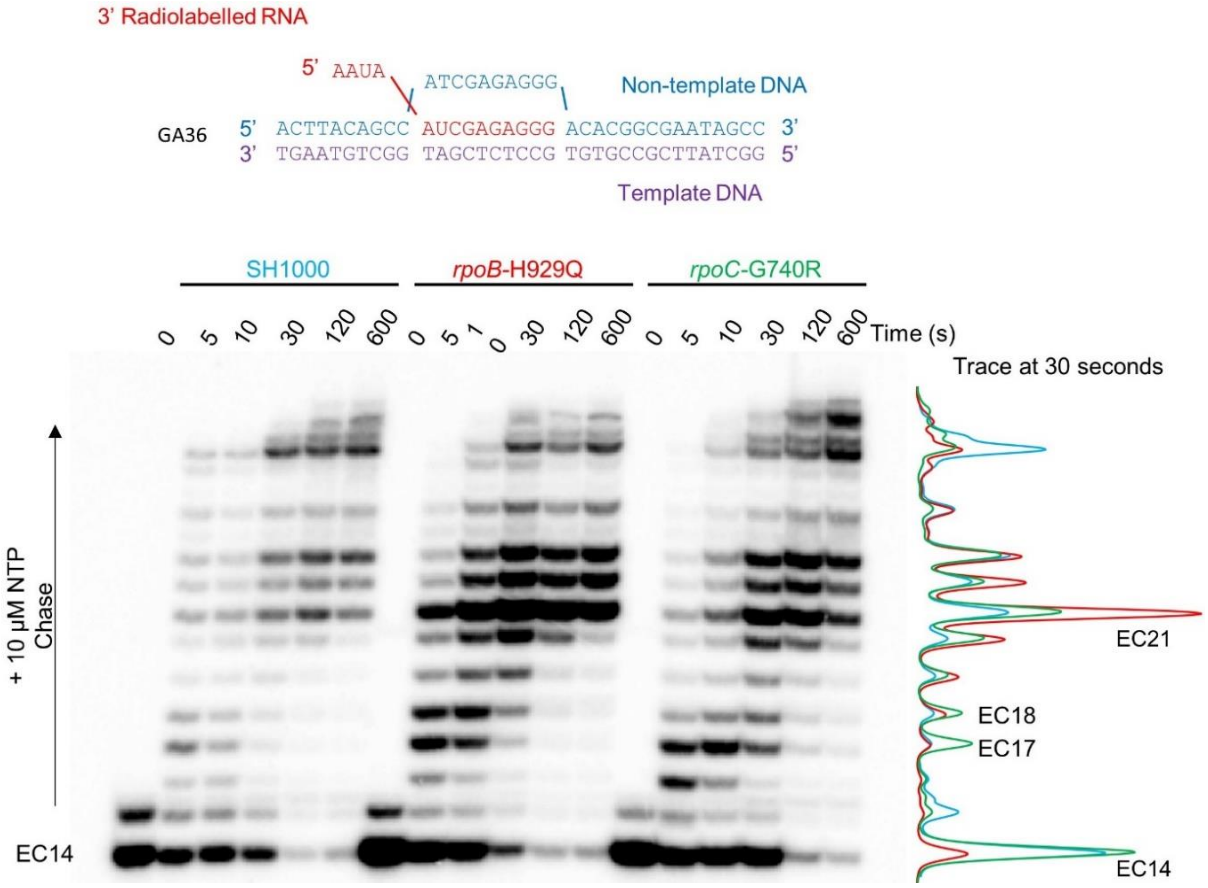

S3 Figure: Transcription elongation and pausing of SH1000, *lysA*::p*mecA rpoB*-H929Q and *lysA*::p*mecA rpoC*-G740R RNAPs.

The assembled scaffold was used to examine differences in pausing by three RNAPs during elongation. The trace of each reaction at 30 seconds on the 23% w/v polyacrylamide denaturing gels can be seen.
