# Supplementary material for: Evolving MRSA: High-level β-lactam resistance in Staphylococcus aureus is associated with RNA Polymerase alterations and fine tuning of gene expression
Source: PLoS Pathog. 2020 Jul 24;16(7):e1008672. doi: 10.1371/journal.ppat.1008672 (PMC7380596; doi:10.1371/journal.ppat.1008672)
Supplement: S4 Fig — RNA of was radiolabelled on the 5’ end to produce an RNA of 13 nt. Misincorporation resulted in the production of an RNA product of 15 nt. A) Schematic of scaffold before and after misincorporation. B) 23% w/v polyacrylamide denaturing gel showing misincorporation over time. Observed rate constants (Kobs) are shown below the gel (numbers that follow the ± sign represents standard errors). (PDF) [file ppat.1008672.s012.pdf]

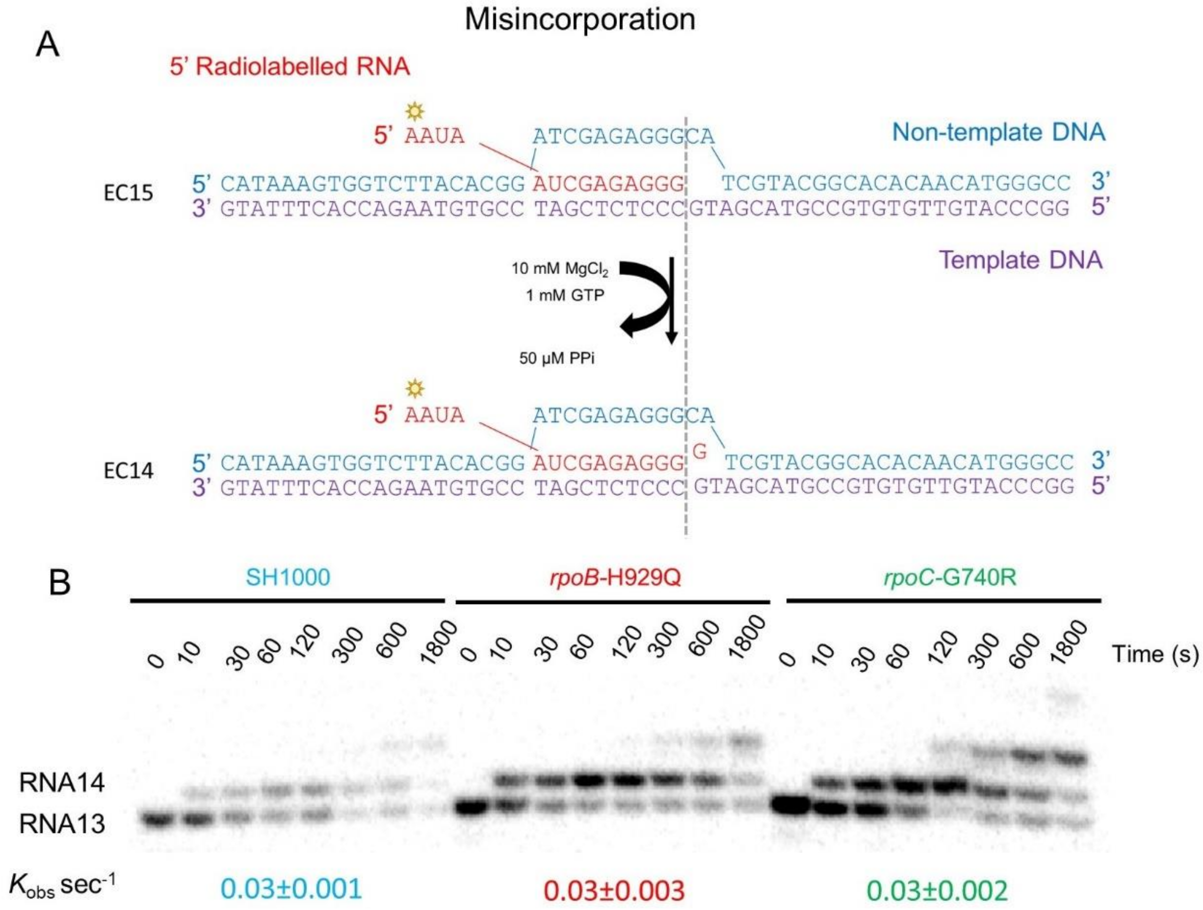

S4 Figure: Misincorporation by SH1000, *lysA*::p*mecA* *rpoB*-H929Q and *lysA*::p*mecA* *rpoC*-G740R.

RNA of was radiolabelled on the 5' end to produce an RNA of 13 nt. Misincorporation resulted in the production of an RNA product of 15 nt. **A)** Schematic of scaffold before and after misincorporation. **B)** 23% w/v polyacrylamide denaturing gel showing misincorporation over time. Observed rate constants (K_{obs}) are shown below the gel (numbers that follow the ± sign represents standard errors).
