# Supplementary material for: Evolving MRSA: High-level β-lactam resistance in Staphylococcus aureus is associated with RNA Polymerase alterations and fine tuning of gene expression
Source: PLoS Pathog. 2020 Jul 24;16(7):e1008672. doi: 10.1371/journal.ppat.1008672 (PMC7380596; doi:10.1371/journal.ppat.1008672)
Supplement: S5 Fig — RNA was radiolabelled on the 5’ end to produce an RNA of 15 nt. Hydrolysis resulted in the production of a radiolabelled RNA product of 13 nt. A) Intrinsic cleavage of mis-incorporated nucleotide by RNAPs; (I) Schematic of scaffold before and after intrinsic hydrolysis. (II) 23% w/v polyacrylamide denaturing gel showing hydrolysis over time. Observed rate constants (Kobs) are shown below the gel (numbers that follow the ± sign are standard errors). B) Gre factor-associated hydrolysis of mismatched nucleotide by RNAPs. Gre-assisted hydrolysis resulted in the production of a radiolabelled RNA product of 13 nt. (I) Schematic of scaffold before and after hydrolysis. (II) 23% w/v polyacrylamide denaturing gel showing hydrolysis over time. Observed rate constants (Kobs) are shown below the gel (numbers that follow the ± sign are standard errors). (PDF) [file ppat.1008672.s013.pdf]

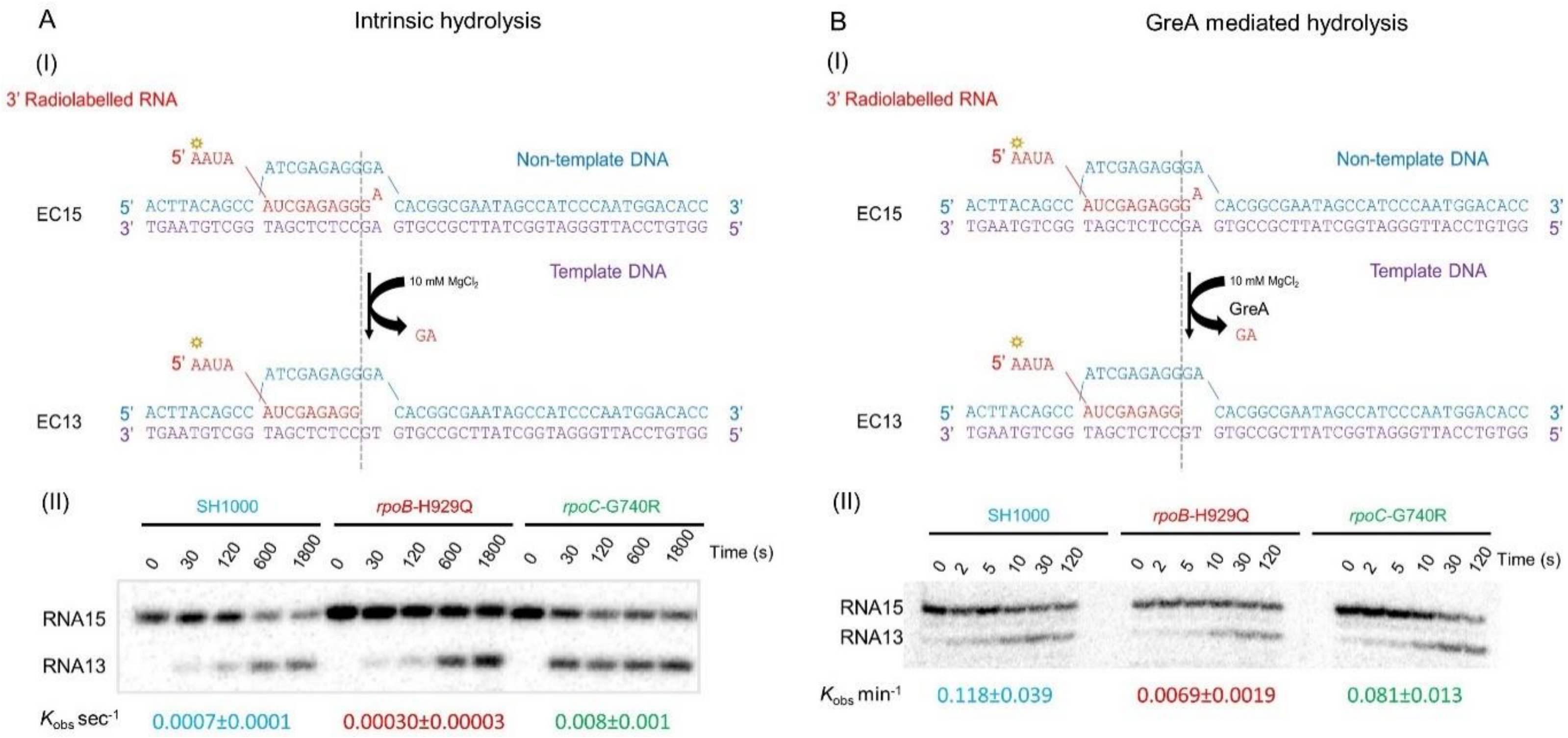

S5 Figure: RNA hydrolysis of a non-cognate nucleotide by SH1000, *lysA*::p*mecA rpoB*-H929Q and *lysA*::p*mecA rpoC*-G740R.
RNA was radiolabelled on the 5' end to produce an RNA of 15 nt. Hydrolysis resulted in the production of a radiolabelled RNA product of 13 nt. **A)** Intrinsic cleavage of mis-incorporated nucleotide by RNAPs; (I) Schematic of scaffold before and after intrinsic hydrolysis. (II) 23% w/v polyacrylamide denaturing gel showing hydrolysis over time. Observed rate constants (K_{obs}) are shown below the gel (numbers that follow the ± sign are standard errors). **B)** Gre factor-associated hydrolysis of mismatched nucleotide by RNAPs. Gre-assisted hydrolysis resulted in the production of a radiolabelled RNA product of 13 nt. (I) Schematic of scaffold before and after hydrolysis. (II) 23% w/v polyacrylamide denaturing gel showing hydrolysis over time. Observed rate constants (K_{obs}) are shown below the gel (numbers that follow the ± sign are standard errors).
