# Supplementary material for: Evolving MRSA: High-level β-lactam resistance in Staphylococcus aureus is associated with RNA Polymerase alterations and fine tuning of gene expression
Source: PLoS Pathog. 2020 Jul 24;16(7):e1008672. doi: 10.1371/journal.ppat.1008672 (PMC7380596; doi:10.1371/journal.ppat.1008672)
Supplement: S6 Fig — The terminator (Term), run off (R/O) and abortive products produced after 30 min of T7A1 incubation with the RNAPs were visualised on a 20% w/v polyacrylamide denaturing gel. (PDF) [file ppat.1008672.s014.pdf]

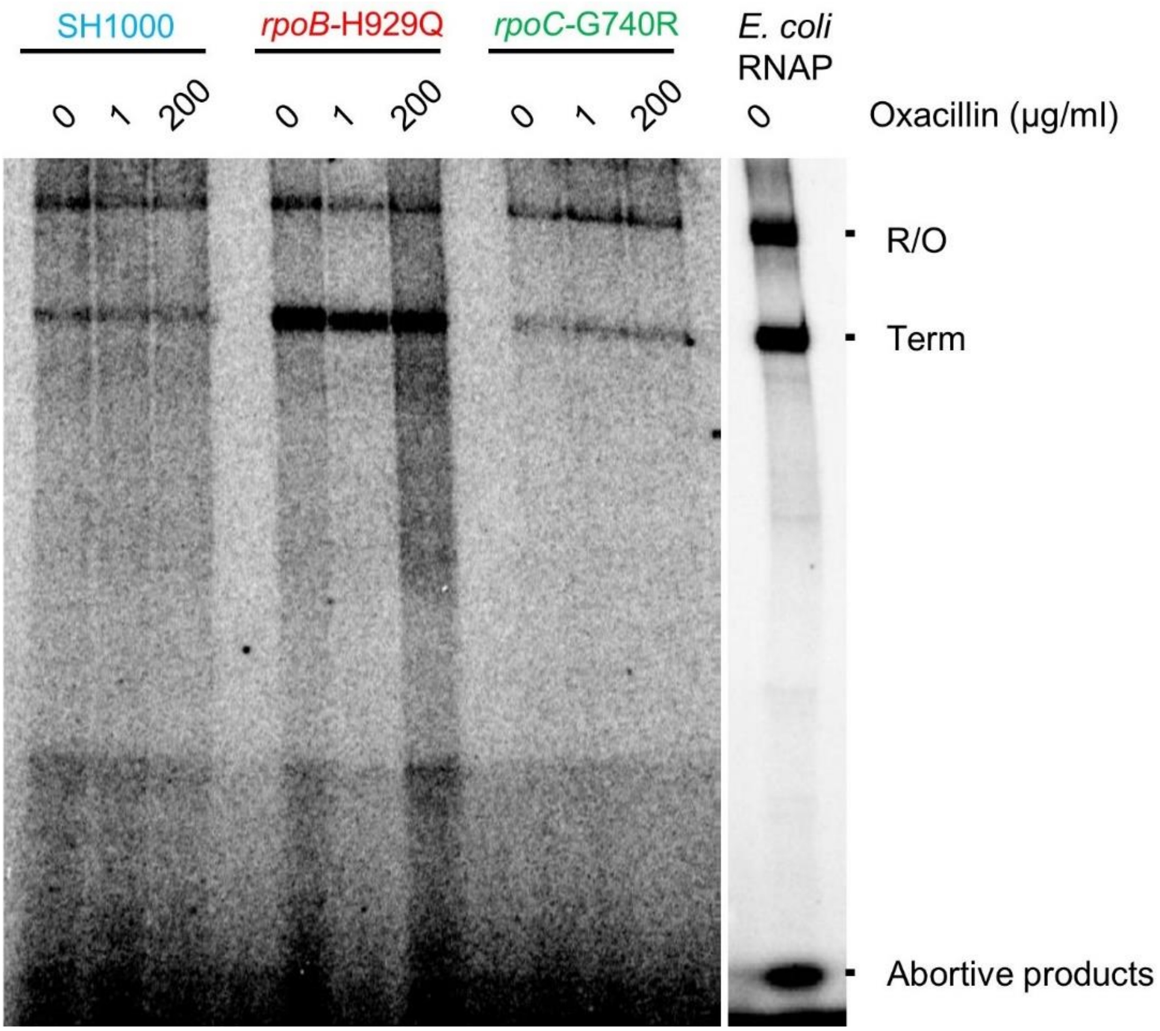


**S6 Figure: Transcription of SH1000, *lysA*::p*mecA rpoB*-H929Q and *lysA*::p*mecA rpoC*-G740R on T7A1 in the presence and absence of oxacillin at high and low concentrations.**

The terminator (Term), run off (R/O) and abortive products produced after 30 min of T7A1 incubation with the RNAPs were visualised on a 20% w/v polyacrylamide denaturing gel.
