# Supplementary material for: Evolving MRSA: High-level β-lactam resistance in Staphylococcus aureus is associated with RNA Polymerase alterations and fine tuning of gene expression
Source: PLoS Pathog. 2020 Jul 24;16(7):e1008672. doi: 10.1371/journal.ppat.1008672 (PMC7380596; doi:10.1371/journal.ppat.1008672)
Supplement: S7 Fig — A) Schematic representation of antibiotic gradient plate of two layers. Bottom layer consists of plain BHI agar, top layer supplemented with 5/20 μg/ml methicillin. B) Resistance properties of pRB474-pmecA (SJF4981) and its parental MSSA, SH1000 strain. C) Use of a gradient plate to select for high-level oxacillin resistance. D) The Etest strips revealed high-level oxacillin resistance which required presence of the pRB474-pmecA. (PDF) [file ppat.1008672.s015.pdf]

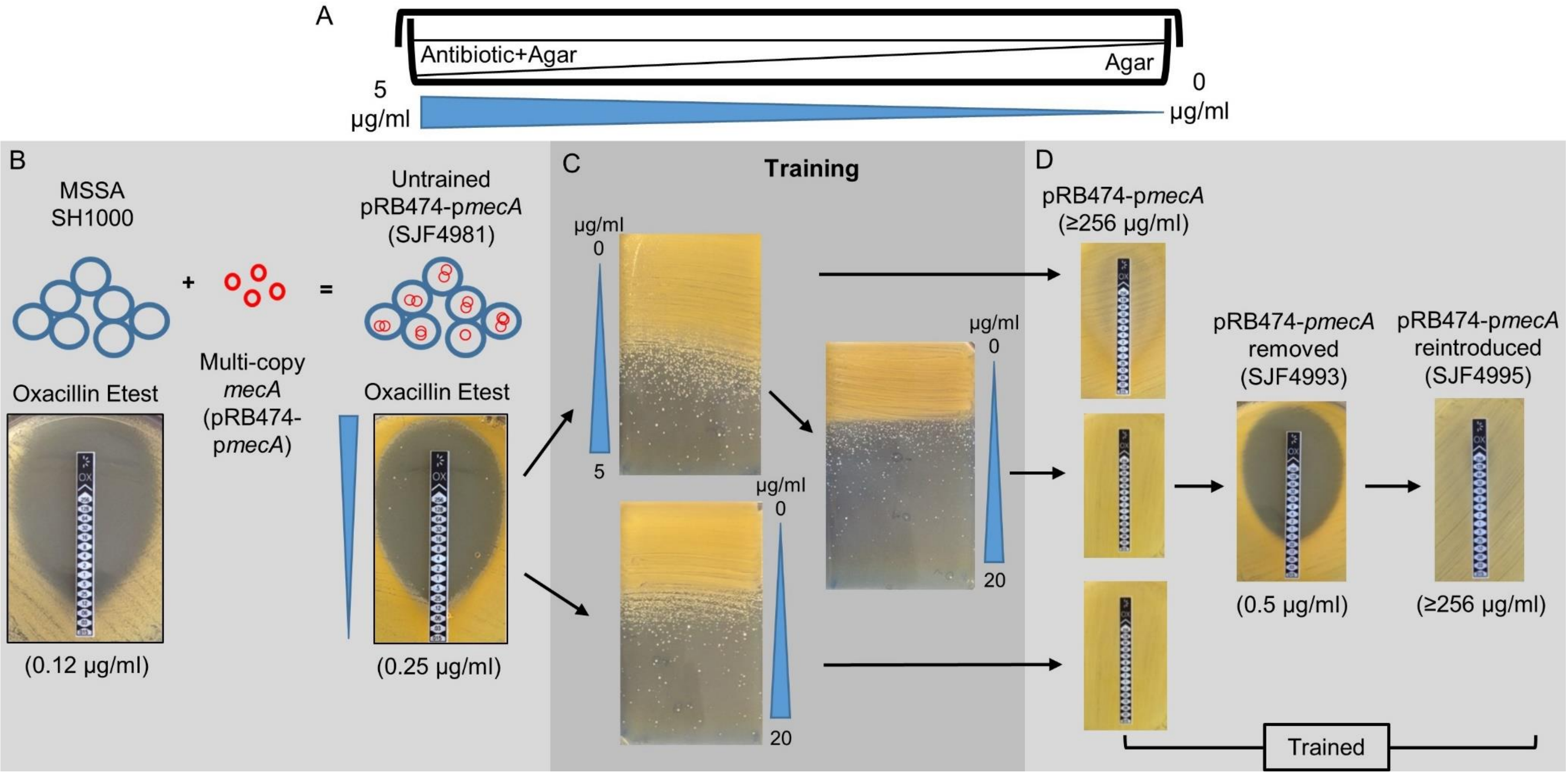

S7 Figure: Schematic representation of high-level MRSA selection using multicopy plasmid-borne *mecA* and subsequent strain evolution.

A) Schematic representation of antibiotic gradient plate of two layers. Bottom layer consists of plain BHI agar, top layer supplemented with 5/20 µg/ml methicillin. **B)** Resistance properties of pRB474-p*mecA* (SJF4981) and its parental MSSA, SH1000 strain. **C)** Use of a gradient plate to select for high-level oxacillin resistance. **D)** The Etest strips revealed high-level oxacillin resistance which required presence of the pRB474-p*mecA*.
