# Supplementary material for: Evolving MRSA: High-level β-lactam resistance in Staphylococcus aureus is associated with RNA Polymerase alterations and fine tuning of gene expression
Source: PLoS Pathog. 2020 Jul 24;16(7):e1008672. doi: 10.1371/journal.ppat.1008672 (PMC7380596; doi:10.1371/journal.ppat.1008672)
Supplement: S8 Fig — A) The genomic region of the gdpP operon along with rplI and dnaC genes. The N-terminus of the GdpP protein contains two transmembrane helices (black boxes), a PAS domain, GGDEF, DHH and DHHA1 domains. Amino acid substitutions identified in highly resistant derivatives of pRB474-pmecA (SJF4981) are indicated and strain details are shown in boxes. B) The inactivation of gdpP in ΔgdpP::kanR (SJF5025) showed susceptibility to oxacillin in the absence of mecA. Subsequent introduction of pRB474-pmecA into ΔgdpP::kanR (SJF5025) resulted in ΔgdpP::KanR pRB474-pmecA (SJF5026) was accompanied by high-level resistance to oxacillin. The MIC for oxacillin determined by Etest is listed in brackets. (PDF) [file ppat.1008672.s016.pdf]

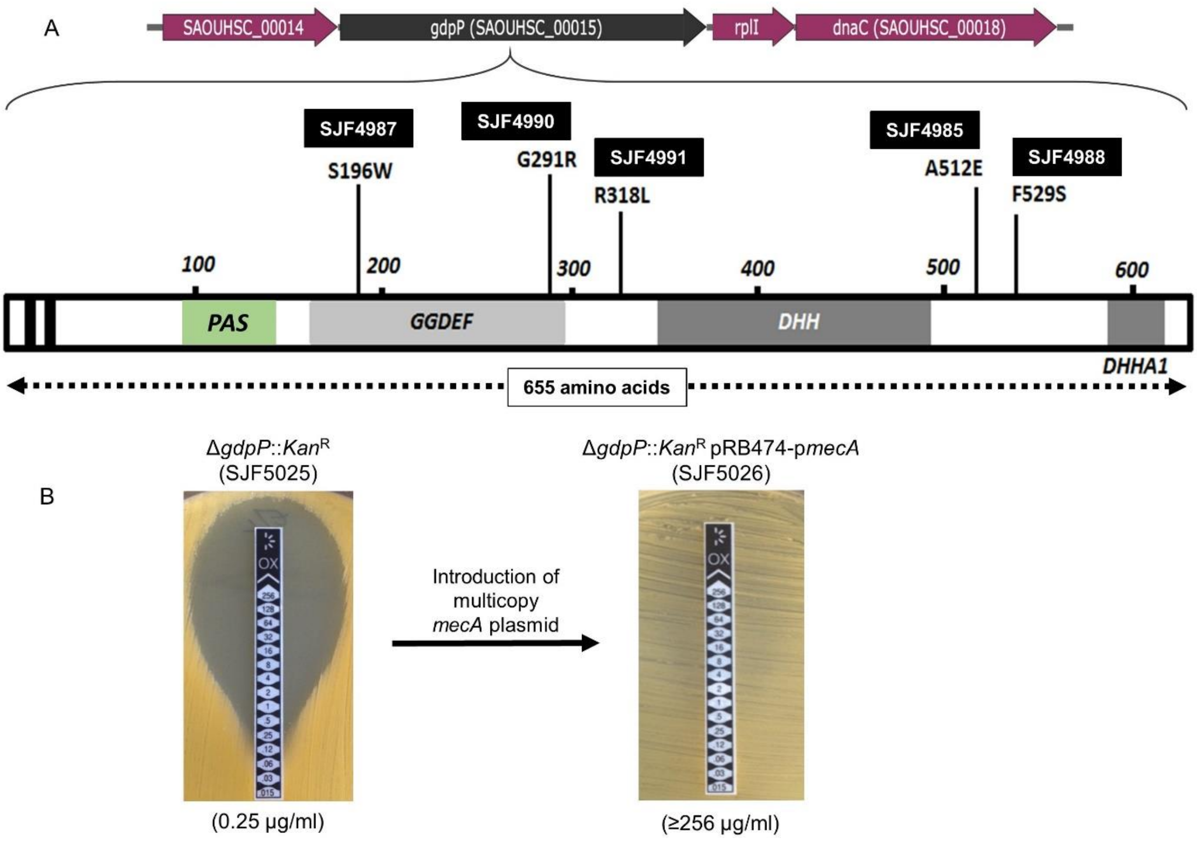

S8 Figure: Schematic of the GdpP operon, showing the acquired SNPs and effect of *gdpP* deletion on resistance.

A) The genomic region of the *gdpP* operon along with *rplI* and *dnaC* genes. The N-terminus of the GdpP protein contains two transmembrane helices (black boxes), a PAS domain, GGDEF, DHH and DHHA1 domains. Amino acid substitutions identified in highly resistant derivatives of pRB474-p*mecA* (SJF4981) are indicated and strain details are shown in boxes. **B)** The inactivation of *gdpP* in Δ*gdpP*::*kan*^{R} (SJF5025) showed susceptibility to oxacillin in the absence of *mecA.* Subsequent introduction of pRB474-p*mecA* into Δ*gdpP*::*kan*^{R} (SJF5025) resulted in Δ*gdpP*::*Kan*^{R} pRB474-p*mecA* (SJF5026) was accompanied by high-level resistance to oxacillin. The MIC for oxacillin determined by Etest is listed in brackets.
