# Supplementary material for: Evolving MRSA: High-level β-lactam resistance in Staphylococcus aureus is associated with RNA Polymerase alterations and fine tuning of gene expression
Source: PLoS Pathog. 2020 Jul 24;16(7):e1008672. doi: 10.1371/journal.ppat.1008672 (PMC7380596; doi:10.1371/journal.ppat.1008672)
Supplement: S9 Fig — A) gdpP-R318L (SJF4993) was transduced with single copy mecA using RN4220 lysA::pmecA (SJF4994) as a donor strain for the chromosomal integration of pmecA into the multicopy mecA (pRB474-pmecA) cured background. B) Vice versa, lysA::kan rpoB-H929Q (SJF5010) was introduced with multicopy plasmid-borne pRB474-pmecA into the single copy mecA cured background. Oxacillin MICs are listed in brackets for all strains. (PDF) [file ppat.1008672.s017.pdf]

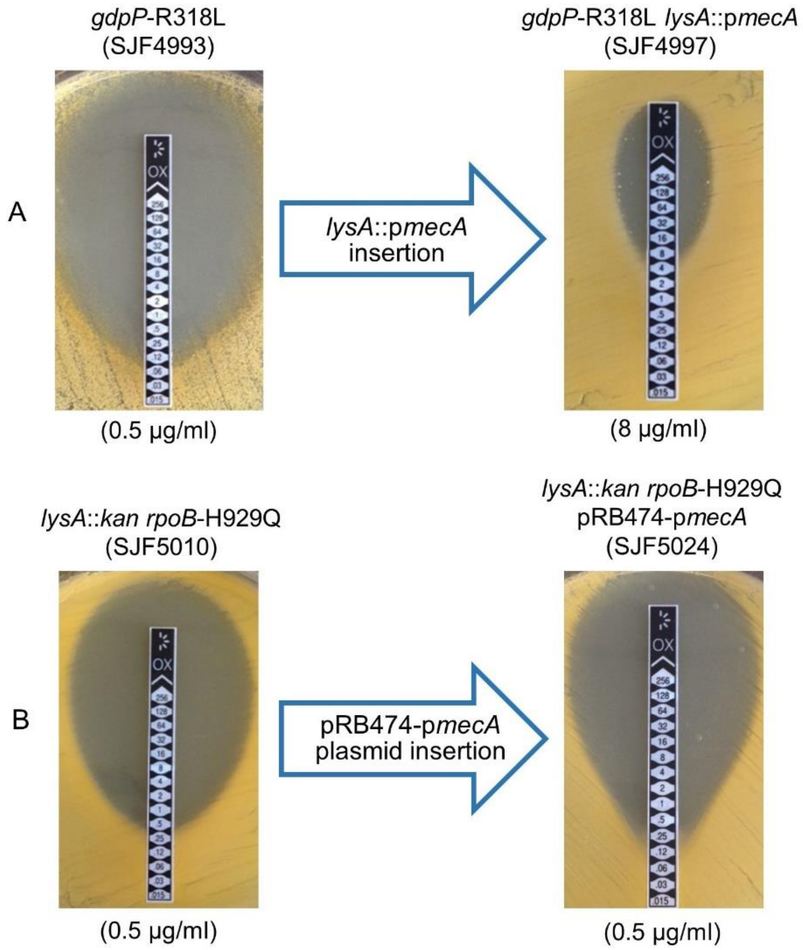

S9 Figure: Reintroduction of *mecA* into p*mecA* cured backgrounds.

A) *gdpP*-R318L (SJF4993) was transduced with single copy *mecA* using RN4220 *lysA*::p*mecA* (SJF4994) as a donor strain for the chromosomal integration of p*mecA* into the multicopy *mecA* (pRB474-p*mecA*) cured background. **B)** Vice versa, *lysA*::*kan rpoB*-H929Q (SJF5010) was introduced with multicopy plasmid-borne pRB474-p*mecA* into the single copy *mecA* cured background. Oxacillin MICs are listed in brackets for all strains.
