# Supplementary material for: Evolving MRSA: High-level β-lactam resistance in Staphylococcus aureus is associated with RNA Polymerase alterations and fine tuning of gene expression
Source: PLoS Pathog. 2020 Jul 24;16(7):e1008672. doi: 10.1371/journal.ppat.1008672 (PMC7380596; doi:10.1371/journal.ppat.1008672)
Supplement: Attachment — Submitted filename: Response to Reviewers_V6.docx [file ppat.1008672.s018.docx]

**Editor’s Comments:**

*Please provide additional functional data and reorganize and rewrite parts of the manuscript to improve its readability as suggested by the reviewers.*

We have addressed the reviewer’s comments in full (see below). In particular, we have analysed the roles of multiple components in high level antibiotic resistance and have carried out pathogenesis studies to determine the effect of resistance in disease. The manuscript has also been substantially edited to make it more accessible to the reader.

**Reviewer's Responses to Questions**

**Part I - Summary**

*Reviewer #1: However, much of the data is descriptive and although a number of genes are implicated in the high level resistance phenotype associated with the rpo mutations, their precise contribution to the resistance phenotype remains unknown.*

To determine the role of identified genes in antibiotic resistance (*mecA* and *rpo* associated) a further mutagenesis study has been carried out. In total the functions of 14 genes were determined. Of all the single mutations tested only an unmarked deletion in *SAOUHSC_00271* led to a decreased MIC for oxacillin. The identified gene is part of the Type VII secretion system and alludes to a role for this mechanism in resistance. This will be the subject of future studies. A new section has been added to the manuscript to include this data (l. 373-387).

*Reviewer #2: The authors also note the presence of an rpoC mutation, R857H, in USA300 (table S4). However, it should be noted that this strain lineage is a highly heterogeneous in its expression of methicillin resistance, so this would not seem to entirely fit with the rpoB/C mutations as facilitating homogeneous resistance; this might be worth a minor comment as to why.*

It is very interesting that USA300 FPR3757 has an *rpoC* mutation and yet it is quite sensitive to oxacillin. This could be due to another mutation elsewhere in the chromosome which could render oxacillin susceptibility. A statement to this effect is now included in the text (l. 174-177).

*In terms of general comments, the text is extremely difficult to follow at times given the large number of mutant constructs, trained and untrained, etc, and whatever can be done to reinforce reminders and remind the reader of what exactly is going on would be welcome.*

We agree that the plethora of strains can make things difficult to follow. We have now simplified the strain names throughout the manuscript. The full provenance of each strain is described in Table S7.

*In addition, the experiments were conducted within the context of no regulators (mecR1-mecI or blaR1-blaI), one or both of which are almost always present in naturally occurring strains. This is a potential limitation of the generalizability of the data from these experiments.*

There are a variety of regulatory regimes, for instance COL with SCC*mec* type I has a truncated regulatory system, resulting in *mecA* constitutive expression. Pre-MRSA N315 with SCC*mec* type II has a regulatory system and interestingly upon acquisition of *rpoB* exhibits high-level resistance to beta-lactams. MRSA252 also has SCC*mec* type II as well as a SNP in *rpoB* and *rpoC* and associated resistance to beta-lactams. We cannot claim the *rpo* association to be universal but the mutations certainly occur in a variety of strains.

*Minor comments.

1. Lines 68-72 read: Methicillin-resistant S. aureus (MRSA) strains are not only resistant to virtually all β-lactam antibiotics but also other classes of antibiotics. This is due to the ability of MRSA to shift from being low- to high-level resistant. Prior studies revealed the involvement of chromosomal mutations for the transition of resistance but the underlying mechanism is still unknown. This is a bit misleading in that the shift from low to high-level resistance is in reference to beta-lactams not other antibiotic classes.*

This has been corrected to make the correlation only with beta-lactams and not other classes of antibiotics (l. 52-53).

*2. The failure of plasmid-borne mecA to confer high level resistance in the single copy pmecA cured strain (SJF5010) is interesting. Information needs to be included about the promoter for mecA on the plasmid. It is not clear why this would be the case and that the means by which the copy number and vector of mecA would select for different mechanisms is not obvious and perhaps worthy of comment.*

This section has now moved in the manuscript as requested by Reviewer 3 and is there as a comparator to the single copy *mecA* (l. 273-296). In the plasmid version *mecA* is under the control of its own promoter (l. 275-276). It is certainly interesting that the plasmid and single copy versions leading to high level resistance have independent mechanisms (l. 282-288). We hypothesised that this might be due to PBP2A levels but this does not seem to be the case (l. 289-296).

*3. Table S4. USA300 is a clone type, not a strain. Please indicate which strain. Also, the abbreviations (OX, FOX, PG, RIF) lack annotation. RIF is obvious, as is OX, but the others may not be. Presumably PG = penicillin G, which if this is the case, the USA 300 strain seems to have lost its beta-lactamase plasmid and thus its regulators.*

Table S4 is now moved in the manuscript to Table S2. The USA300 strain name has been corrected and the abbreviations are now annotated (Table S2).

**Part II – Major Issues: Key Experiments Required for Acceptance**

Reviewer #1:

*1. The authors have identified several genes that may be important for expression of high level resistance in strains carrying rpo mutations. To validate the study conclusions, however, it would be helpful to demonstrate experimentally that one or more of these genes is required for expression of high level resistance. For example can the high level resistance phenotype be altered by genetically manipulating expression / function of one or more of these genes?*

We have analysed the role of several of the identified genes in high level resistance (see response to Part I Reviewer #1).

*2. The authors have done some nice experiments with COL, showing that restoration of an rpoB mutations renders this strain oxacillin susceptible (Fig. S4). Given the focus of this journal on pathogenesis and in order to further investigate the clinical significance of these data, the authors should investigate the virulence of their highly resistant strains carrying rpo mutations in comparison to the wild type strain and untrained strain carrying single mecA. Is there a change in virulence of the rpo-repaired COL strain? Many highly resistant hospital MRSA strains are relatively less virulent that MSSA or CA-MRSA strains. What are the implications of the data reported in this study for virulence?*

We agree that this is a very important implication for our manuscript. In response we have carried out a comprehensive *in vivo* analysis of the pathogenesis of our matched strains using the murine sepsis model. Seven strains were compared and the results are shown in Figure 9 with associated methods (l. 639-653), results (l. 388-400) and discussion (l. 450-457). The strains compared were in the SH1000 and COL backgrounds. The results show that, in the SH1000 background, the presence of *mecA* actually increases both kidney colonisation and weight loss. Importantly there are no apparent fitness costs associated with high level resistance. Thus, using our defined strains there is no overriding effects on virulence due to the acquisition of resistance *per se*.

*Reviewer #3: • The manuscript is a bit confusing and I believe that it needs to be reorganized in a simple way. For instance, the manuscript starts with the strains complemented with mecA in the multicopy plasmid. However, the rest of the manuscript is dedicated to investigate the strains with the chromosomal copy of mecA. I believe that the originality of the manuscript comes from the approach of using strains with a single chromosomal copy of mecA. Thus, I will suggest to remove the results obtained with the strains complemented with the plasmid.*

As suggested we have removed the plasmid-based *mecA* from the beginning of the manuscript to makes things easier to follow. The importance of the *mecA* plasmid comes in being able to control the level of PBP2A and to correlate this with resistance. We have therefore included this comparative analysis later on (l. 273-296) so as not to detract from the single copy *mecA* work.

*• The abstract of the manuscript is very confusing and long. It needs to be rewritten completely.*

The abstract has been redrafted for clarity.

*• Fig 3C The authors conclude that rpoB-H929Q was impaired in initiating transcription from the SigA-dependent promoters whilst it behaved similarly at the SigB-dependent asp23 promoter. The results are not as clear because rpoB-H929Q still showed a significant inhibition in transcription initiation of asp23 promoter.*

Figure 3C is now Figure 3A. We agree that it is difficult to make firm conclusions as to the effects on RNAP activity. The run off and abortive synthesis transcription assays show the activity of the specific preparation of purified RNAP. The elongation assays, which do not depend on specific activity of a particular preparation, showed that all RNAP were equally active but had different properties in various elongation reactions (S3 Figure).

This characterisation of RNAPs was aimed at showing whether the mutations lead to direct alteration of RNAPs’ properties (as opposed to, for example, alteration of interaction with transcription factors). The results indeed show such alterations. However, dissecting the mechanisms of how these altered properties of mutant RNAPs are linked to the high level of methicillin resistance is outside of the scope of our study.

As the analysis of WT and mutant RNAPs properties *in vitro* was merely to determine the functionality of the mutations in *rpo*, we changed the text to downplay the conclusions from the *in vitro* experiments (l. 428-435).

*• Line 463. Regulation of gene expression in methicillin-resistant strains is largely accounted by downregulation of Rex regulator. Thus, complementation with Rex should result in the repression of Rex-regulated genes. Does complementation with Rex reduce the resistance to methicillin of trained RpoB-H929Q, trained pmecA cured rpoB-H929Q, and trained rpoC-G740R strains? is a Rex mutant resistant to methicillin?*

To determine the role of *rex*, we analysed the effect of a *rex* mutation on high level resistance as part of a functionality study (see response to Part I Reviewer #1). The *rex* mutant did not have an altered oxacillin MIC.

*• RpoBC mutants specifically affect the expression of certain genes (11 genes including mecA). According to the results, it is suggested that transcription pausing plays a major role in this regulation. It would be important to analyze using reporter genes fused to some of the promoters that the there are no differences in the transcription initiation.*

As mentioned above (3^rd^ response, Part II, Reviewer #3), we used characterisation of RNAPs *in vitro* to determine if mutations in RNAPs have direct effect on RNAP properties, which turned out to be the case. Dissecting the molecular mechanisms of these effects (whether initiation or termination are affected and how such effects relate to defects in elongation) and how it links to methicillin resistance is a large project for the future. We have downplayed conclusions from *in vitro* experiments to reflect this uncertainty.

*• An important unanswered question is why strains producing rpoC-G740-R that has low transcription initiation have increased expression of mecA in vivo. It would be important to measure the mRNA levels by northern or RT-PCR and the levels of the transcription initiation by fusing the promoter of mecA to a reporter gene.*

The transcription initiation assays (Fig 3A) show the activity of the specific preparation of purified RNAP. It cannot be concluded that a mutant RNAP is less or more active than WT RNAP, because the fraction of active RNAP in this particular preparation can be lower than for WT. Dissecting the molecular mechanisms of how *rpo* mutations lead to differential expression – and if it is initiation, elongation, termination, or all of them – is the matter of a future extensive study. In the present study, the message is that the expression of some genes is altered (as seen from RNA-seq), and the reason for that is likely the mutations in RNAP as these mutations change catalytic properties of RNAPs (as seen in elongation assays).

**Part III – Minor Issues: Editorial and Data Presentation Modifications**

*Reviewer #1: The authors should make the manuscript results section more concise and focused. The data with the plasmid-borne mecA is not very novel (gdpP mutations associated with high level resistance have been reported before) and could be streamlined or moved to supplementary allowing the manuscript to focus more on the integrated mecA strains carrying rpo mutations. It would also be helpful to find a more accessible naming system for the strains. At present the reader has to work quite hard to follow the complicated strain names. Similarly, the results section describing the transcriptional profiling is quite long and streamlines or parts moved to supplementary data.*

As suggested, we have moved, and streamlined, the plasmid mediated *mecA* part of the work to allow a focus on the single copy version. We have now simplified the strain names throughout the manuscript, with a full description in Table S7. Finally, the section on transcriptional profiling has been cut down to make things easier to follow.

*Reviewer #2: The paper is extremely difficult to read and follow, at least for me, and supposedly I actually know the field. Whatever the authors and editors can do to simplify and clarify the message would be welcome.*

We have given the manuscript a thorough overhaul to simplify it for ease of reading.

*Reviewer #3: •* *Line 210. I cannot find mutations in SAOUHSC_00591 in Table S4.*

The mutations are in Table S2 (previously Table S4) associated with strains SJF5000, SJF5003 and SJF5004.

*• Lines 258. Revised the text “…only applicable to the SH1000 strain a clinical MSSA isolate S. aureus Newman [37] was…”*

This has been revised for clarity (l. 190-192).

*• Line 323. Figure 3A is describe latter than Fig. 3C. Panels of Fig. 3 should be easily reorganized to follow the description of the results in the text.*

The order of Figure 3 has now been changed to follow the description.

*• In general, it would be helpful for the reader to maintain the name of the strains all along the manuscript, for example, “Trained lysA::pmecA rpoB-H929Q (SJF5003)” is name as “Trained rpoB-H929Q” in the last section of the manuscript.*

We have simplified the strain designations and made them consistent throughout. All strains are fully described in Table S7.
