# Supplementary material for: Evolving MRSA: High-level β-lactam resistance in Staphylococcus aureus is associated with RNA Polymerase alterations and fine tuning of gene expression
Source: PLoS Pathog. 2020 Jul 24;16(7):e1008672. doi: 10.1371/journal.ppat.1008672 (PMC7380596; doi:10.1371/journal.ppat.1008672)
Supplement: Attachment — Submitted filename: Response to Reviewer Comments_V8.docx [file ppat.1008672.s019.docx]

**Editor’s comments:**

*Modify the manuscript according to the review recommendations.*

**Reviewer's Responses to Questions**

**Part I - Summary**
*Please use this section to discuss strengths/weaknesses of study, novelty/significance, general execution and scholarship.


Reviewer #1: The authors have improved the clarity and readability of the revised manuscript and added new data. However, while it is acknowledged that the manuscript contains a substantial amount of data, the study remains largely descriptive and does not substantially advance previous studies indicating that rpo mutations are associated with high level beta-lactam resistance in MRSA.*

*Reviewer #2: PPATHOGENS-D-19-01601 reports results of experiments designed to identify the genetic basis for expression of high levels of resistance (i.e., higher MICs) to oxacillin by mutants selected by passage of a methicillin-susceptible SH1000 parent strain (termed “training”) into which mecA has been introduced on a multi-copy plasmid or as a single copy with its native promoter into the chromosome. Introduction of mecA in “untrained” recipients only slightly increased toe MIC compared to the susceptible parent and the transformants phenotypically were susceptible. “Training” of these transformant by selection on methicillin greatly increased their MICs. Interestingly, the mutations associated with training of the SH1000 transformants were different depending on whether mecA was on a multi-copy plasmid or integrated as a single copy into the chromosome. When these trained mutants were cured of mecA and then mecA was re-introduced on the same vector as originally used, MICs were fully restored. On the other hand, if mecA was re-introduced in the cured, trained mutants with the other vector, resistance was not restored or only partially restored. The authors conclude that genetic backgrounds matter, but the contribution of how mecA is delivered also seems to be important and should not be dismissed. This phenomenon is not investigated further, as the investigators appropriately turn their attention to the more biologically relevant transformants containing a single copy of mecA with its native promoter, however, it might be worth a quick mention in the discussion. The results clearly demonstrate a key role of rpo mutations in trained mutants containing a single mecA gene. This is confirmed in another strain, Newman, and in a panel of clinical MRSa strains, past and extant. A plausible explanation of how these rpo mutations might confer high level resistances is provided by results of other experiments, although the fine details need to be worked out. One factor not investigated is the role of beta-lactamase and mecR1-mecI regulatory genes which are present in naturally occurring strains but not in strains used in these experiments. This might be commented upon. Overall, these experiments provide new insights into the phenotypic resistance expressed by MRSA strains and the molecular and biochemical basis for it.*

As suggested, we have added a comment to reflect the importance of *mecA* delivery route in the mechanism of resistance (l. 291-293).

The potential effects of the range of regulatory variants and the presence of beta-lactamase is now commented on (l. 462-464).

*1. It is not entirely clear from the results whether introduction of plasmid mecA or chromosomal mecA into SH1000 was not accompanied by any mutations besides those present on the plasmid vectors prior to “training.” This seems to be the case, but worth explicitly stating. Unless the parent and untrained mecA+ strains were not sequenced, it is not possible to conclude that the key mutations in rpo genes and gdpP were the result of antibiotic selection.*

We have added text to state that the whole genome sequencing of SH1000, SJF4996 (single copy *mecA*) and SJF4981 (plasmid-borne *mecA*) identified no other mutations than those directly associated with the genetic manipulation (l. 140-142 and l. 279-280).

*2. Is the promoter for mecA in pRB474-pmecA native or otherwise? And if not native, what is the promoter?*

For both versions of strains, we used the native promoter for the expression of *mecA* (l. 119, 277, 480)

*3. The description of results described in lines 144 through 194 is a bit hard to follow. It might be worthwhile to create a figure of a flow diagram showing the genetic manipulations, the mutants, and the MICs.*

We are very aware this is a complicated manuscript and we have been careful to reference all the Figures and Tables as we go through. An extra Figure, we believe would complicate things further.

*4. Please provide methods for MIC determinations. Presumably there were all Etest and no supplemental NaCl in the media.*

Method provided (l. 503-504).

*Reviewer #3: (No Response)*

**Part II – Major Issues: Key Experiments Required for Acceptance**
*Please use this section to detail the key new experiments or modifications of existing experiments that should be absolutely required to validate study conclusions.

Generally, there should be no more than 3 such required experiments or major modifications for a "Major Revision" recommendation. If more than 3 experiments are necessary to validate the study conclusions, then you are encouraged to recommend "Reject".*


*Reviewer #1: The authors refer to a member of the S. aureus Type VII secretion system that is required for high level resistance. This is the most novel aspect of the study and needs to be developed in order to provide new insights on the acquisition of high level beta-lactam resistance in MRSA.*

*Reviewer #2: No major issues

Reviewer #3: (No Response)*

**Part III – Minor Issues: Editorial and Data Presentation Modifications**
*Please use this section for editorial suggestions as well as relatively minor modifications of existing data that would enhance clarity.*


*Reviewer #1: (No Response)

Reviewer #2: Few...articulated in comments to authors

Reviewer #3: (No Response)*
